# A Stable High‐Performance Zn‐Ion Batteries Enabled by Highly Compatible Polar Co‐Solvent

**DOI:** 10.1002/advs.202403513

**Published:** 2024-07-17

**Authors:** Shuo Yang, Guangpeng Wu, Jing Zhang, Yuning Guo, Kui Xue, Yongqi Zhang, Yuanmin Zhu, Tao Li, Xiaofeng Zhang, Liujiang Zhou

**Affiliations:** ^1^ School of Physics State Key Laboratory of Electronic Thin Films and Integrated Devices University of Electronic Science and Technology of China Chengdu 611731 China; ^2^ Institute of Fundamental and Frontier Sciences University of Electronic Science and Technology of China Chengdu 611731 China; ^3^ Research Institute of Interdisciplinary Science & School of Materials Science and Engineering Dongguan University of Technology Dongguan 523808 China; ^4^ Institute of Materials and Physics Ganjiang Innovations Academy Chinese Academy of Sciences Ganzhou 341119 China

**Keywords:** dissolution, highly compatible, low‐temperature, phase transition, polar‐solvents, solvation structure optimization, trifluoroethanol, Zn dendrites

## Abstract

Uncontrollable growth of Zn dendrites, irreversible dissolution of cathode material and solidification of aqueous electrolyte at low temperatures severely restrict the development of aqueous Zn‐ion batteries. In this work, 2,2,2‐trifluoroethanol (TFEA) with a volume fraction of 50% as a highly compatible polar‐solvent is introduced to 1.3 M Zn(CF_3_SO_3_)_2_ aqueous electrolyte, achieving stable high‐performance Zn‐ion batteries. Massive theoretical calculations and characterization analysis demonstrate that TFEA weakens the tip effect of Zn anode and restrains the growth of Zn dendrites due to electrostatic adsorption and coordinate with H_2_O to disrupt the hydrogen bonding network in water. Furthermore, TFEA increases the wettability of the cathode and alleviates the dissolution of V_2_O_5_, thus improving the capacity of the full battery. Based on those positive effects of TFEA on Zn anode, V_2_O_5_ cathode, and aqueous electrolyte, the Zn//Zn symmetric cell delivers a long cycle‐life of 782 h at 5 mA cm^−2^ and 2 mA h cm^−2^. The full battery still declares an initial capacity of 116.78 mA h g^−1^, and persists 87.73% capacity in 2000 cycles at −25 °C. This work presents an effective strategy for fully compatible co‐solvent to promote the stability of Zn anode, V_2_O_5_ cathode and aqueous electrolyte for high‐performance Zn‐ion batteries.

## Graphical abstract

Schematic illustrations of the positive effects of TFEA co‐solvent in full batteries.

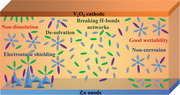

## 1 Introduction

Because of low redox potential (−0.762 V vs. standard hydrogen electrode (SHE)), high volume capacity (5585 mA h cm^−3^), low toxicity, cheapness, and safety, rechargeable aqueous Zn‐ion batteries (ZIBs) are generally considered to have the most potential to replace Li‐metal batteries for large‐scale applications in the field of energy storage.^[^ ^1^, ^2^, ^3^ ^]^ Unfortunately, they are difficult to meet market requirements due to the irreversible dissolution of the cathode, uncontrollable growth of Zn dendrite on the anode and poor ionic conductivity of aqueous electrolyte at low temperatures.^[^ ^4^, ^5^, ^6^, ^7^ ^]^ On one hand, during long‐term cycles, part of the cathode materials such as V_2_O_5_ will dissolve into electrolyte inevitably, leading to significant capacity degradation and ultimately battery failure.^[^ ^8^, ^9^, ^10^ ^]^ More unfortunately, the Zn dendrites accumulated during repeat charge/discharge processes are a stumbling block to developing high‐performance Zn‐based energy storage devices. Excessive growth of Zn dendrites can cause the separator to be punctured, causing internal short circuits in the batteries, thereby compromising the coulombic efficiencies (CEs) during batteries cycling and shortens batteries lifespan.^[^ ^10^, ^11^, ^12^ ^]^ On the other hand, due to the solidification phase transition of aqueous electrolytes in low‐temperature environments, the ionic conductivity sharply decreases, resulting in the loss of ability to store electrical energy in batteries.^[^ ^13^, ^14^, ^15^ ^]^ Obviously, compared to electrode modification at anode side or cathode side, incorporating additives into the electrolyte offers a simultaneous solution to these challenges by effectively impacting the cathode material, Zn anode, and electrolyte.

Electrolyte modification engineering stands out as the most convenient, efficient, and cost‐effective method to address the three challenges simultaneously.^[^ ^16^, ^17^, ^18^, ^19^, ^20^, ^21^ ^]^ One strategy for modifying electrolytes is to introduce salts such as LiCl, TBA_2_SO_4_, and ChCl, which can prevent direct contact between Zn^2+^ and Zn anode, thereby inhibiting the growth of Zn dendrites.^[^ ^22^, ^23^, ^24^, ^25^, ^26^ ^]^ However, those low‐concentration salts are difficult to prevent water from freezing at low temperatures.^[^ ^27^ ^]^ On the contrary, high concentrations of salts will cause serious corrosion to the Zn anode and current collector.^[^ ^13^ ^]^ Some organic solvents and aqueous electrolytes can be mixed in any ratio, forming a co‐solvent electrolyte system that can effectively inhibit water freezing. Meanwhile, researchers have found that diethyl ether and acetonitrile can adjust Zn^2+^ electrodeposition direction by adsorbing onto the anode.^[^ ^28^, ^29^ ^]^ Dimethyl sulfoxide and propylene carbonate have been studied to reshape the solvation structure of Zn^2+^ and form the in‐situ solid electrolyte interface.^[^ ^30^, ^31^ ^]^ Furthermore, methanol, as a co‐solvent in 1 M Zn(CF_3_SO_3_)_2_ (Zn(OTf)_2_) electrolyte, can break the hydrogen bonds (H‐bonds) among water molecules and promote uniform deposition of Zn anode, thus broadening the operating‐temperature range and improving the electrochemical performance of full battery.^[^ ^32^ ^]^ It is worth noting that trifluoroacetamide can generate strong interactions with Zn^2+^, leading to changes in the inherent structure of Zn(H_2_O)_6_^2+^ and enhancing the transport of Zn^2+^.^[^ ^33^ ^]^ Meanwhile, the interaction between C═O groups in trifluoroacetamide and H_2_O disrupts the original H‐bonding network structure in water, thereby decreasing the activity of H_2_O and increasing the low‐temperature stability of the electrolyte. Those effects should be attributed to the enhanced polarity of the C═O group by C−F. In addition, N,*N*‐dimethylformamide has been demonstrated the ability to anchor into the layered structure of the cathode material, thereby improving the cyclic stability of the full battery.^[^ ^34^, ^35^ ^]^ Obviously, these previous studies prove that the organic co‐solvents are potential optimal candidates for simultaneously dealing with the key issues on the cathode, Zn anode, and electrolytes.^[^ ^36^, ^37^, ^38^, ^39^ ^]^ Unfortunately, there are few reports of co‐solvents that demonstrate positive effects on the three components in batteries concurrently. Therefore, the development of electrolyte co‐solvents with high compatibility with cathode, anode, and electrolyte is crucial for the large‐scale application of secondary aqueous ZIBs.

Herein, we developed an effective, low‐cost, and weakly‐acidic hybrid electrolyte containing 1.3 M Zn(OT)_2_, 2,2,2‐trifluoroethanol (TFEA, volume fraction: 50%) and water, which is applied to high‐performance V_2_O_5_//Zn full batteries suitable for low temperatures. Initially, numerous theoretical calculations were conducted to uncover the advantages of TFEA in comparison to other polar solvents. Then, we found that the optimal volume fraction of TFEA in the electrolyte is 50%, which possesses excellent cold‐resistance (Freezing point less than 75.49 °C) and ionic conductivity (18.49 mS cm^−1^) simultaneously. The electrochemical performance of Zn//Zn symmetric cells, Cu//Zn half‐cells, and V_2_O_5_//Zn full batteries systematically verified the positive effects of TFEA on Zn anode, electrolyte, and V_2_O_5_ cathode, meaning the high compatibility of TFEA. Finally, the role mechanisms of TFEA on those components in full batteries were carefully proven through various theoretical calculations and advanced characterization.

## 2 Results and Discussion

Nowadays, common monohydric alcohols, such as methanol, ethanol, and isopropanol etc., have been used in ZIBs successfully.^[^ ^40^, ^41^, ^42^, ^43^, ^44^, ^45^, ^46^, ^47^ ^]^ Those organic alcohol solvents are miscible with aqueous electrolytes due to their polar hydrophilic group (−OH). Therefore, those alcohols with −OH can be regarded as the classical polar co‐solvents capable of modifying the H‐bonds network of water molecules and reshaping the solvated structure of Zn^2+^, thus enhancing the low‐temperature performance of electrolytes and promoting the uniform Zn deposition.^[^ ^48^ ^]^ Besides, the nonpolar hydrophobic group (−CH_3_ and −CH_2_−) possessed by those alcohols cannot effectively disrupt the H‐bonds between water molecules.^[^ ^49^ ^]^ Therefore, when the carbon chain of a monohydric alcohol is too long, the solution undergoes stratification (Figure S1, Supporting Information). Subsequent investigations reveal that the electrostatic potential distribution (ESP) of a single alcohol molecule exhibits strong polarity at the −OH side and non‐polarity at the carbon chain side (Figure S2, Supporting Information). Additionally, as depicted in Figure S3a (Supporting Information), the material density of water (1.00 g cm^−3^) is significantly larger than that of butanol (0.81 g cm^−3^), while the pKa value and dipole moment, indicative of the molecular polarity, do not change significantly (Figure S3b,c, Supporting Information).

**Figure 1:**
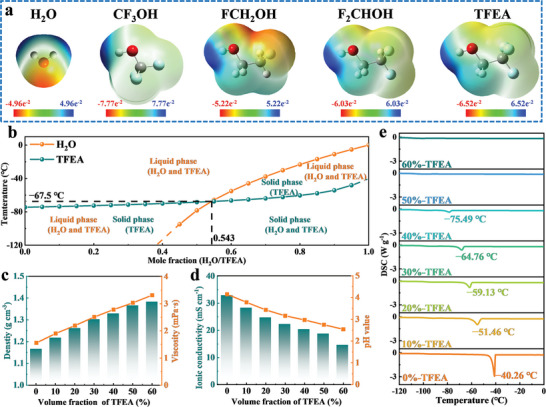
a) Distribution diagrams of electrostatic potential (ESP) of common solvent molecules. b) Phase diagram of water‐TFEA solution. c,d) Various physical properties and differential scanning calorimeter (DSC) curves e) of electrolytes including TFEA with different volume fractions.

In general, the stronger polarity the hydrophilic −OH group shows, the easier it is to form H‐bonds with water molecules.^[^ ^50^, ^51^ ^]^ The introduction of C−F bonds can help increase the polarity of −OH, thereby enhancing its effect of forming H‐bonds with water molecules and inhibiting the solidification of the electrolyte at low temperatures.^[^ ^52^ ^]^ Figure S4 (Supporting Information) shows that those solutions containing 1,1,1‐trifluoro‐2‐propanol, 2,2,3,3,3‐pentafluoropropanol, 4,4,4‐trifluorobutanol, and 2,2,3,3,4,4,4‐heptafluorobutanol still appear to be stratified. The introduction of C−F bonds results in larger absolute values of the electrostatic potentials, larger material density and dipole moment, and lower pKa values, implying that the electron‐withdrawing C−F bonds enhance the polarity of −OH. Moreover, the density surge of those fluoro‐alcohols due to the large number of C−F bonds remains the main cause of solution stratification (**Figure** **1**a; Figure S5, Supporting Information). By the action of the C−F bonds, weakly‐acidic 2,2,2‐trifluoromethanol (CF_3_OH) has the strongest acidity (The ionization degree of −OH group (Ka) is 10^−7.65^) (Figure S6, Supporting Information). However, CF_3_OH is not suitable for the operating environment of ZIBs because its −OH group is very unstable and prone to oxidation during the charging process.^[^ ^53^, ^54^ ^]^ Meanwhile, the most significant density difference exists between 1,1,1,3,3,3‐hexafluoroisopropanol (1.596 g cm^−3^) and water (1 g cm^−3^), which may cause instability of the aqueous electrolyte during operation. Among all these fluoro‐alcohols, TFEA has relatively moderate density (1.393 g cm^−3^), excellent water solubility (soluble with water in any proportion) and stable chemical property, as well as strong polarity (dipole moment: 3.38 D, pKa: 12.19). More importantly, compared with other fluoro‐alcohols, such as 2‐fluoroethanol (FCH_2_OH), 2,2‐difluoroethanol (F_2_CHOH), etc., TFEA cosolvents are more affordable (only 0.07 USD g^−1^) due to the more simply synthesis process (Table S1, Supporting Information). Furthermore, as shown in the simulated phase diagram in Figure 1b, the mixed water‐TFEA solution has a low freezing point (−67.5 °C) when the molecular ratio of H_2_O to TFEA is 0.543:0.457, indicating better low‐temperature resistance. Therefore, TFEA has been selected as an ideal polar co‐solvents to achieve the mass destruction of the H‐bonds network in water and reshape the Zn^2+^ solvated structure.

To further identify the optimal ratio of TFEA and validate the role of TFEA on aqueous Zn‐based electrolytes, various physical properties of electrolytes containing 1.3 M Zn(OTf)_2_ and different volume fractions of TFEA (denoted as X%‐TFEA, where X represents the volume fractions of TFEA) are shown in Figure 1c,d. With volume fractions increasing, both the material density and viscosity gradually rise, leading to a progressive decline in the ionic conductivity. In addition, the pH value of 60%‐TFEA decreases to 2.54, attributed to lower pKa of TEFA compared to water, thus providing more H^+^. Admittedly, the decrease in pH value caused by an increase in H^+^ concentration can exacerbate hydrogen evolution corrosion of Zn anode.^[^ ^55^ ^]^ The optical images of the Zn//Zn symmetric cells at the initial stage and quieting for 20 days are shown in Figure S7 (Supporting Information). Obviously, the two symmetrical cells do not experience any bulging or deformation, indicating that the hydrogen evolution corrosion in 0%‐TFEA and 50%‐TFEA can be ignored. The provided scanning electron microscopy (SEM) images and X‐ray diffraction (XRD) patterns of the Zn anodes after soaking for 20 days are shown in Figures S8 and S9 (Supporting Information). Both Zn anodes exhibit some holes caused by corrosion, meaning that the hydrogen evolution corrosion by electrolyte is inevitable. Moreover, compared to 0%‐TFEA, the holes distribution of Zn anode in 50%‐TFEA is more uniform. The XRD patterns of those Zn anodes are similar to the initial Zn foil, implying that the Zn corrosion by electrolyte is acceptable. Even if the pH value of 50%‐TFEA drops to 2.75, it does not cause severe natural corrosion reaction on Zn anode, indicating that the Zn anode can tolerate the acidic environment with 50%‐TFEA. This is mainly because the change in pH value caused by TFEA is relatively small, and the H^+^ concentration is still low. Furthermore, the SEM images of Zn anodes soaking in strong acidic electrolytes are shown in Figure S10 (Supporting Information). In the red cycles, a severe hydrogen evolution corrosion on the Zn anode after soaking in 0%‐TFEA + 0.01 M CF_3_SO_3_H appears in Figure S10a (Supporting Information). However, in Figure S10b (Supporting Information), TFEA can significantly inhibit the hydrogen evolution corrosion caused by CF_3_SO_3_H, thus reducing the reaction activity of free H^+^ by forming H‐bonds between −OH and H^+^.^[^ ^27^, ^48^ ^]^

Meanwhile, the DSC curves illustrate a gradual decrease in the electrolyte's freezing point with increasing volume fraction, accompanied by a diminishing intensity of the heat‐absorption peak (Figure 1e). Notably, the freezing point of 40%‐TFEA reaches as low as −75.49 °C, while the heat absorption peak disappears entirely when further increasing the volume fraction of TFEA to 50%. Considering that continuing to increase the volume fraction will cause the pH and ionic conductivity to be lower, thus leading to a decrease in the performance of the battery, we proceeded to utilize 50%‐TFEA modified electrolyte to further investigate its electrochemical performance due to the suitable pH (2.75) and conductivity (18.79 mS cm^−1^), and good resistance (The freezing point is lower than −75.49 °C) for the working environment of ZIBs.

**Figure 2:**
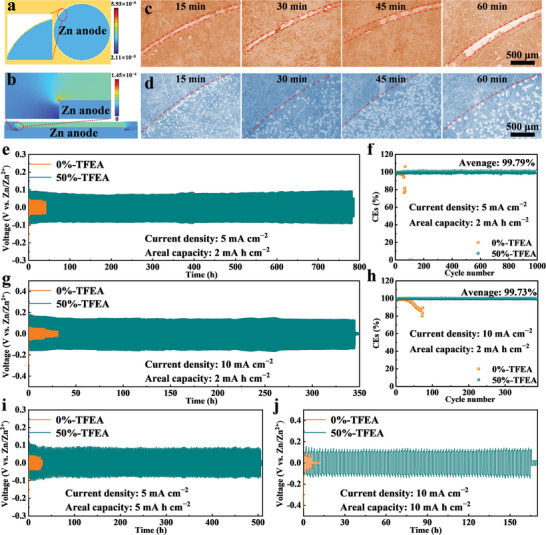
a) Charge density and b) electric field strength of the button cell electrode by finite element analysis. SEM images of Zn anode with non‐woven separator at different electrodeposition times in 0%‐TFEA c) and 50%‐TFEA d) at 2 mA cm^−2^. Long‐term galvanostatic curves of Zn//Zn symmetrical cells and Cu//Zn half cells at e,f) 5 mA cm^−2^ and g,h) 10 mA cm^−2^. Galvanostatic curves of Zn//Zn symmetrical cells under depth discharge conditions (i) 5 mA h cm^−2^ and j) 10 mA h cm^−2^).

As well known, due to charge edge distribution, the tip effect of electrode is the main reason for the uncontrolled growth of the Zn dendrites. Specifically, compared with the smooth part, the surface charge density at the tip part is higher and the electric field strength near the tip part is stronger, meaning that it is easier to discharge or charge from the tip to the electrolyte. More importantly, a stronger electric field is generated at the tip of Zn dendrites, which attracts more Zn^2+^ toward the tip, thus leading to further Zn deposition on the dendrites. However, the Zn foil has been polished before being used, so there are no obvious dendrites on the surface in the beginning. As shown in **Figure** **2**a,b, when a button cell operates normally, the charges accumulate at the arc of Zn anode, creating a stronger electric field compared to the central area. Due to the charge accumulation, it is easier to drive Zn^2+^ to the edge, leading to more severe growth of Zn dendrites. For the above reason, Zn dendrites in 0%‐TEFA occur almost entirely on the arc of the Zn anode and then gradually become wider and larger as the deposition time increases (Figure 2c). Fortunately, this phenomenon has been completely suppressed after adding the co‐solvent (Figure 2d). With the increase of deposition time, a lot of fine Zn nuclei clearly not only exist at the edges but also appear at the center area of the Zn anode, suggesting that TFEA may have an electrostatic shielding effect on the edges of the Zn anode and promote the uniform nucleation of the Zn anode. The SEM images of Zn anodes with glass‐fiber separator after deposition in 0%‐TFEA and 50%‐TFEA are shown in Figure S11 (Supporting Information). Figure S11a,d (Supporting Information) show the surface of the Zn anode without deposition. After a deposition of 10 mA cm^−2^ for 1 h, the Zn anode in 0%‐TFEA exhibits distinguishable areas of non‐deposition and deposition (Figure S11b,e, Supporting Information). There is a clear stacking of dendrites in the deposition area, and those dendrites are embedded with glass fibers from the separator. On the contrary, the Zn anode in 50%‐TFEA exhibits a more uniform distribution of Zn deposition without any obvious bugles, indicating a higher space utilization (Figure S11c,f, Supporting Information). Under the same Zn deposition amount (10 mA h cm^−2^), the active area of Zn deposition of 50%‐TFEA is significantly larger than that of 0%‐TFEA. This means that a more uniform deposition surface with smaller and shorter Zn dendrites will be formed on the anode in 50%‐TFEA.

Moreover, as shown in Figure 2e, the Zn//Zn symmetric cell without TFEA has short‐circuited after 32 h of normal operation at a current density of 5 mA cm^−2^. By contrast, after introducing TFEA, the symmetric cell can be able to operate stably for 783 h, more than 24 times longer than before. Under the same charge/discharge condition, the CEs of Cu//Zn half‐cell using aqueous electrolyte quickly experience an intense shock at the 60th cycle, due to uncontrollable growth of Zn dendrites (Figure 2f). On the contrary, the half‐cell with hybrid electrolyte can run stably for more than over 1000 cycles. Even when the current density increases to 10 mA cm^−2^, the symmetric cell still has a long cycle‐life of up to 340 h (Figure 2g,h). Also, the half‐cell works stably for 390 cycles with average CEs as high as 99.73%, implying that TFEA can remarkably inhibit the generation of dead Zn even under a high‐current operating condition, which promotes the reversibility of deposition/stripping of Zn. Furthermore, under the charge/discharge areal capacities of 5 mA h cm^−2^ and 10 mA h cm^−2^, the cycle lives of the symmetric batteries with 50%‐TFEA are 17.94 times and 24.34 times longer than those with 0%‐TFEA, respectively, proving that 50%‐TFEA is suitable for operating under higher depth discharge conditions (Figure 2i,j). Table S2 (Supporting Information) shows the cycling performance of Zn//Zn symmetric cells with various modified electrolytes. The Zn//Zn symmetric cell with only 1.3 M Zn‐based electrolyte exhibits an acceptable inhibition effect on Zn dendrites by TFEA, which presents a certain competitiveness.

**Figure 3:**
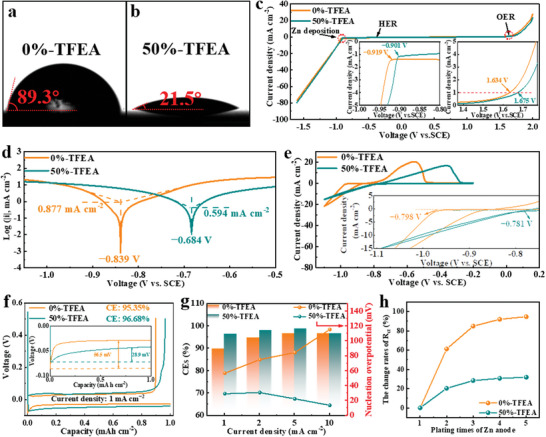
a,b) Contact angle test on Zn anode with electrolytes. c) Linear sweep voltammetry (LSV) curves of electrolytes by a three‐electrode device (The reference electrode is saturated calomel electrode (SCE), the counter electrode and working electrode are stainless steel foils). d) Tafel curves of Zn anode and e) cyclic voltammetry (CV) curves of Cu foil. f) Charge–discharge curves of Cu//Zn half cells at 1 mA cm^−2^ and g) nucleation overpotentials at 1–10 mA cm^−2^. h) Change rates of *R*_ct_ of Zn anode at different plating times.

To find out the root cause of the beneficial effects of TFEA on Zn deposition, a sessile drop contact angle technique was used to investigate the wettability of 0%‐TFEA and 50%‐TFEA. Obviously, 0%‐TFEA presents a contact angle of 89.3° on the Zn anode, whereas the angle reduces to 21.5° in 50%‐TFEA solution, signifying that TFEA can incredibly increase the wettability, thereby improving the nucleation area (**Figure** **3**a,b). In fact, the standard hydrogen evolution potential (SHE, −0.242 V vs. SCE) is much higher than the equilibrium potential of Zn (−0.760 V vs. SHE and −1.002 V vs. SCE). Fortunately, both electrolytes show no obvious hydrogen evolution reaction (HER) in Figure 3c. Moreover, the response current attributed to oxygen evolution reaction (OER) in the LSV curve with TFEA is smaller than that in 0%‐TFEA, showing the inhibition effect on the reaction activity of H_2_O. More notably, under the premise of excessive H^+^, TFEA can effectively inhibit HER and OER, which should be related to TFEA inhibiting the reactivity of water molecules by H‐bonds. Meanwhile, there is a significant turning point at −0.995to −1.055 V vs. SCE in the response currents, meaning the initial potential for Zn deposition, which is consistent with Figure 3c (Figure S12, Supporting Information). As shown in Figure 3d, the Tafel plot shows that Zn anode has a higher corrosion potential of −0.684 V vs. SCE and a lower corrosion current of 0.594 mA cm^−2^ in 50%‐TFEA, demonstrating that TFEA can also alleviate the corrosion influence on Zn anode.

The CV curves of Cu//Zn half cells with the two electrolytes are shown in Figure 3e. There are only two distinct reduction and oxidation peaks in the CV curves, which are attributed to the depositing and stripping of Zn. Furthermore, there are no other peaks in both electrolytes, revealing that TFEA does not participate in other side‐reactions during the cell operation. In the insets of Figure 3e, the deposition potential of Zn increases by 17 mV in the modified electrolyte, in agreement with the trend observed in LSV curves. At the 1^st^ charge‐discharge cycle, the Cu//Zn half‐cell with the modified electrolyte exhibits lower nucleation overpotential of 28.9 mV and higher CEs of 96.68%, implying that TFEA can remarkably promote uniform Zn nucleation during the electroplating process and inhibit the generation of dead Zn during the stripping process at 1 mA cm^−2^ current density (Figure 3f). When the current densities increase to 2, 5, and 10, those half‐cells with TEFA also show more excellent electrochemical performance (Figure 3g; Figure S13a,b, Supporting Information). Significantly, compared with 0%‐TFEA, the high current density causes intense 2D diffusion of Zn^2+^, leading to an increase in overpotential on the charge‐discharge curve of 50%‐TFEA in Figure S13c (Supporting Information).^[^ ^56^, ^57^, ^58^ ^]^ This further proves that the adsorption effect of TEFA increases the diffusion energy barrier of Zn^2+^ near Zn dendrites, thus inhibiting the 2D diffusion of Zn^2+^ and suppressing the vigorous growth of dendrites. The electrochemical impedance spectroscopy data verify that the change rate of charge‐transfer resistance (*R*_ct_) of the Zn anode in 0%‐TFEA is 94.67% after 5 deposition times, which significantly declines compared with the *R*_ct_ of the 1st deposition (Figure 3h; Figure S14, Supporting Information). Apparently, the Zn anode influenced by TFEA presents a smaller change rate of *R*_ct_ (31.83%), implying that the physicochemical properties of Zn anode are closer to the initial state, suggesting a flatter electrolyte‐electrode interface in the modified electrolyte.

**Figure 4:**
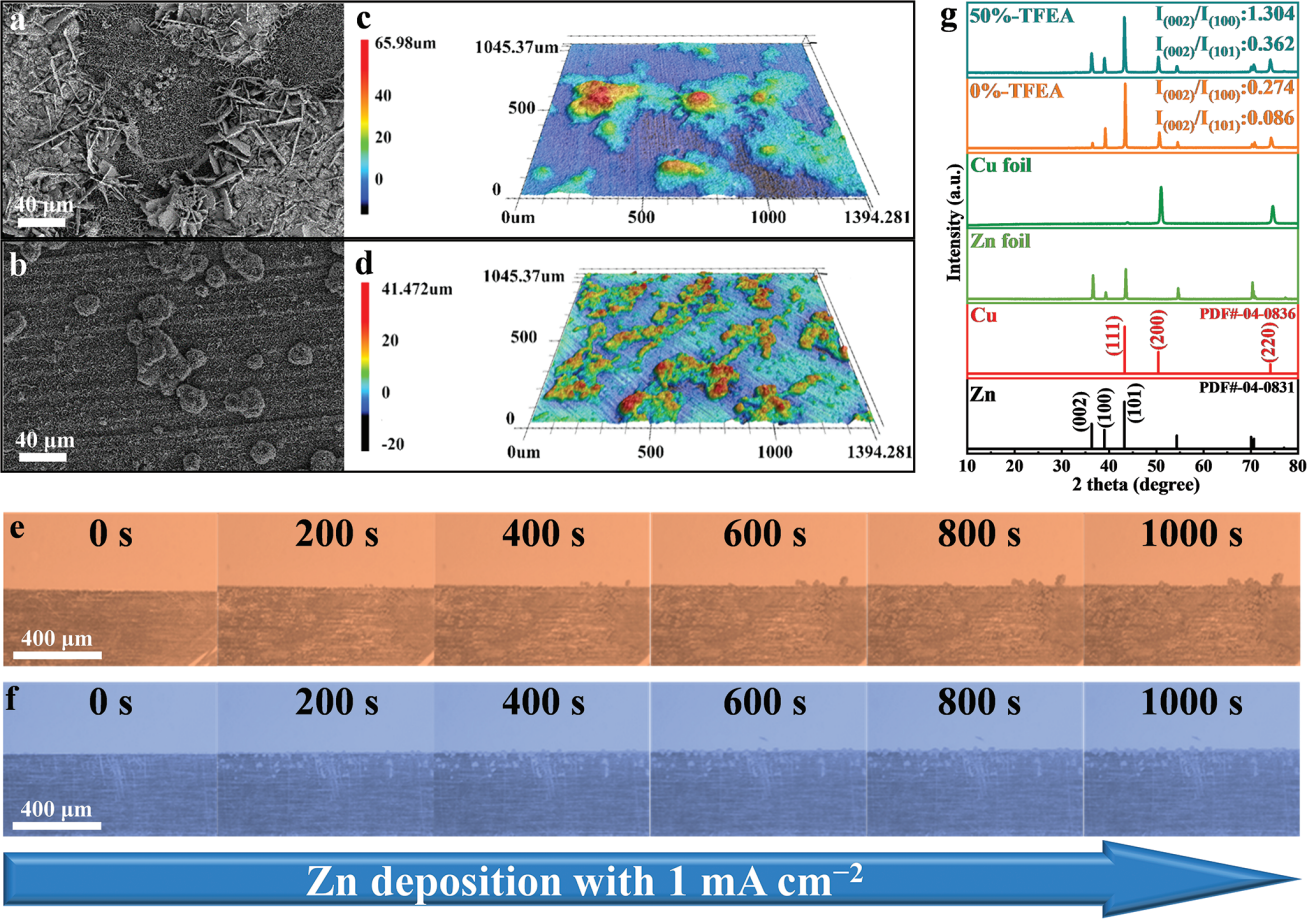
SEM and laser confocal microscope (LSCM) images of Zn anode in a,c) 0%‐TFEA and b,d) 50%‐TFEA at 5 mA cm^−2^ for 1 h. e) XRD of Zn anode at the same test condition. In‐situ optical microscopy images of Zn anode in f) 0%‐TFEA and g) 50%‐TFEA during the plating process at different times.

To intuitively reflect the influence of TFEA on the morphology and component of Zn dendrite during the deposition process, SEM, LSCM, and XRD were applied to analyze as shown in **Figure** **4**a–e. In 0%‐TFEA, the presence of numerous randomly scattered hexagonal Zn single crystals on the Cu foil creates significant voids, leading to diminished space utilization, thus causing uncontrolled growth of Zn dendrites (Figure 4a). In contrast, 50%‐TFEA has a denser surface, and the resulting Zn dendrites are duller and smaller (Figure 4b). Moreover, compared to 0%‐TFEA, the Zn dendrites in the modified electrolyte have a more uniform distribution with lower heights, also consistent with the SEM images (Figure 4c and d). Numerous studies have demonstrated that the (002) crystal plane can promote uniform Zn deposition, while too many (001) or (101) crystal planes will lead to serious growth of dendrites.^[^ ^59^ ^]^ In Figure 4e, the peak intensity ratios (I_(002)_/I_(100)_: 1.304, I_(002)_/I_(101)_: 0.362) in 50%‐TFEA is much higher than that in 0%‐TFEA, which coincides with the excellent cycling performance of symmetric and half‐cells with TFEA above (Figure 2e–h).

To observe the mechanism of Zn dendrite formation directly in the two electrolytes, in‐situ optical microscopy was used to confirm the positive effect of the co‐solvent on the Zn anode (Figure 4f,g). At the first 200 deposition seconds, almost no Zn nuclei are produced on the surface of the Zn anode in 0%‐TFEA, since the main Zn deposition direction is on the cross‐section due to the tip effect. Unacceptably, with the increasing deposition time, the Zn dendrites gradually extend deeper into the electrolyte, and become slenderer and sharper. On the contrary, many white particles appear on the surface of the Zn anode in 50%‐TFEA, implying that Zn deposition occurs not only on the cross‐section but also on the surfaces further away from another electrode. Those Zn particles become progressively larger but always remain unsharp as the deposition time goes on. As a result, the larger area can effectively accommodate Zn produced during deposition thus preventing short‐circuiting of the cell due to excessive deposition in a narrow area, which is consistent with the results of the SEM images and the nucleation overpotential results above (Figure 2c,d and Figure 3f–h).

**Figure 5:**
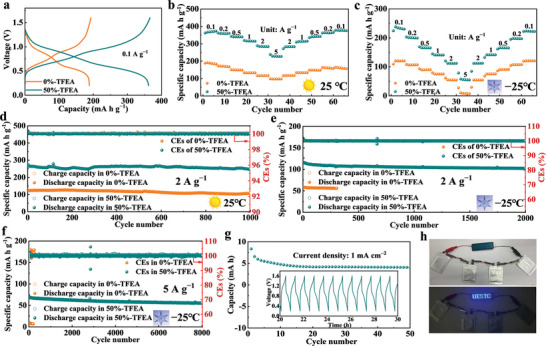
a) Charge–discharge curves at 0.1 A g^−1^ and rate performances at b) 25 °C and c) −25 °C of V_2_O_5_//Zn batteries. Cycle performances of V_2_O_5_//Zn batteries at d) 25 °C and e,f) at −25 °C. g) Cycle performances of a pouch cell at 1 mA cm^−2^. h) Optical photograph of a red lighten light‐emitting diode lighted by four pouch cells.

Due to the excellent theoretical capacity (585 mA h g^−1^) and good tolerance in weakly‐acidic electrolytes, commercialized V_2_O_5_ has been widely used as a universal cathode material in ZIBs.^[^ ^60^, ^61^ ^]^ However, V_2_O_5_ still undergoes irreversible dissolution reactions in electrolytes, making it difficult to maintain a high capacity consistently. Herein, the full batteries with the two electrolytes were further tested to verify the possible positive effect of the co‐solvent on V_2_O_5_ as shown in **Figure** **5**. The V_2_O_5_ cathode in the aqueous electrolyte delivers a specific capacity of 188.53 mA h g^−1^ at 0.1 A g^−1^ (Figure 5a,b). When current density increases to 5 A g^−1^, the cathode only delivers 97.50 mA h g^−1^, which is much lower than that in the hybrid electrolyte (362.09 mA h g^−1^ at 0.1 A g^−1^ and 227.91 mA h g^−1^ at 5 A g^−1^). In Figure 5c, when the test temperature drops to −25 °C, the cathode in 50%‐TFEA can store electrical energy at 5 A g^−1^ (55.42 mA h g^−1^). By contrast, the cathode in 0%‐TFEA is almost unable to function properly. Furthermore, the cycling performance of the battery with 50%‐TFEA at room temperature shows a more stable trend in CEs, demonstrating that TFEA can improve the stability of V_2_O_5_ (Figure 5d; Figure S15, Supporting Information). Even at −25 °C, the batteries with the co‐solvent can run stably for 2000 cycles and 8000 cycles at 2 and 5 A g^−1^, respectively (Figure 5e,f). Moreover, those batteries can still provide 102.28 and 55.56 mA h g^−1^ electrical energy ultimately. The comparison with the reported electrolytes in ZIBs across several performance metrics is shown in Table S3 (Supporting Information). It is obvious that the performance of this work surpasses that of electrolytes with a Zn(OTf)_2_ electrolyte concentration of 1 M at room temperature. Even when compared to other electrolytes with zinc salt concentrations ≥2 M, 50%‐TFEA remains competitively viable (Table S4 and Figure S16, Supporting Information). Moreover, the full battery with only 1.3 M Zn(OTf)_2_ consistently delivers impressive capacity and maintains robust cycling stability at low temperatures, which is extremely rare in similar reports.

The assembled pouch cell can be cycled over 50 cycles at 1 mA cm^−2^ with a capacity of over 4.07 mA h (Figure 5g). The promising application was illustrated to light emitting diode (rated voltage: 4.3 V) with a shape of “UESTC” (Figure 5h). More interestingly, after standing for 100 h, the battery with TFEA can remain 84.64% capacity retention, which is 32.16% higher than that without TFEA (Figure S17, Supporting Information). Moreover, the battery containing TFEA also shows a higher open‐circuit voltage, indicating that TFEA can suppress the interface changes of V_2_O_5_. Although the impedance of the full battery containing TFEA increases to nearly 3000 Ω after 40 h, it gradually stabilizes during 40–100 h, once again reflecting a stable cathode‐electrolyte interface (Figure S18, Supporting Information).

**Figure 6:**
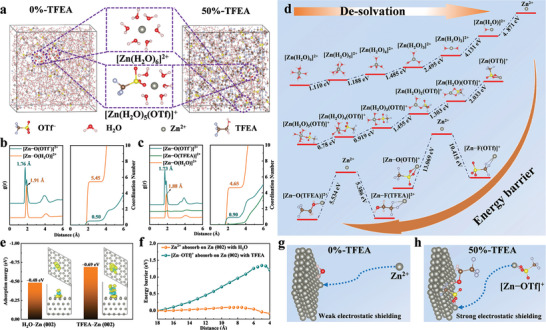
a) Snapshots, b) radial distribution function and c) coordination number by molecular dynamics (MD) simulation. d) Binding energies of various structures of Zn^2+^ calculated by density functional theory (DFT). e) Adsorption energies of a water molecule and a TFEA molecule on the Zn (002) crystal plane. f) Potential energies at a different distance of solvent molecule and g,h) corresponding schematic diagram.

To gain insights into the effect mechanism of the co‐solvent in aqueous electrolyte, MD simulation and DFT calculations were executed to investigate the solvation behavior of Zn^2+^ in TFEA‐containing electrolytes. The MD simulation reveals that the [Zn(H_2_O)_6_]^2+^ and [Zn(H_2_O)_5_OTf]^+^ solvation structures exist in 0%‐TFEA and 50%‐TFEA (**Figure** **6**a). The corresponding radial distribution function and coordination number of Zn^2+^ are shown in Figure 6b,c. The results show that the [Zn‐OTf]^+^ contact ion‐pair in [Zn(H_2_O)_5_OTf]^+^ structure has a shorter coordination distance (1.76 Å) than the [Zn−O(H_2_O)]^2+^ structure (1.91 Å), meaning that OTf^−^ anions is closer to the Zn^2+^ ion because of its negative charge. This tighter encirclement and larger ion volume of OTf^−^ anions can help shield the partial electrical flux from Zn^2+^, thereby preventing the uncontrollable diffusion of Zn^2+^ due to the tip effect. Excitingly, the introduction of TFEA significantly increases the [Zn−(O)OTf]^+^ contact ion‐pair (from 0.50 to 0.90) and reduces the [Zn−O(H_2_O)]^2+^ coordination (from 5.45 to 4.65). On the other hand, the number of H‐bonds between water molecules is greatly reduced by TFEA, indicating an effective inhibition on the solid‐liquid phase transition of the electrolyte at low temperatures (Figure S19a,b and Table S5, Supporting Information). Furthermore, the lower binding energy of (O)TFEA–(H)H_2_O (−0.363 eV) means that TFEA is more likely to form H‐bonds with H_2_O and break the original H‐bonds between (O)H_2_O–(H)H_2_O (−0.247 eV), thus explaining H‐bonds changes in MD simulation (Figure S19c, Supporting Information).

To further evaluate the coordination of Zn^2+^ in the electrolyte more precisely, the energy barriers of various coordination structures were calculated by DFT as shown in Figure 6d. The detailed de‐solvation processes of the [Zn(H_2_O)_6_]^2+^ and [Zn(H_2_O)_5_OTf]^+^ solvation structures from MD results are displayed in Figure 6d. The [Zn(H_2_O)_5_OTf]^+^ structure shows a accumulated de‐solvation energy of 6.49 eV, which is 8.79 eV lower than that of the [Zn(H_2_O)_6_]^2+^ structure, which is more conducive to the nucleation of Zn.^[^ ^32^, ^64^ ^]^ The final de‐solvation structure of [Zn(H_2_O)_5_OTf]^+^ is the contact ion‐pair [Zn−OTf]^+^. The energy barriers of the two coordination forms ([Zn−(O)OTf]^+^ and [Zn−(F)OTf]^+^) of the [Zn−OTf]^+^ structure were also calculated in Figure 6d. Interestingly, The energy barrier of [Zn−(O)OTf]^+^ and Zn^2+^ is 13.069 eV, which is 2.654 eV higher than that of [Zn−(F)OTf]^+^, indicating that Zn^2+^ is more easily coordinated with the O atoms with a negative charge in OTf^−^. Therefore, during the plating process, Zn^2+^ electro‐migrates to the surface of Zn dendrites in the form of contact ion‐pairs under electric field effect in 50%‐TFEA. This is meaning that OTf^−^ anions can follow Zn^2+^ to enrich near Zn dendrites, partly neutralizing the electrostatic field generated by Zn^2+^, thereby weakening the interaction between Zn^2+^ and the negative charges on the dendrite.^[^ ^36^ ^]^ Meanwhile, a similar phenomenon also occurs in [Zn−(O)TFEA]^2+^ and [Zn−(F)TFEA]^2+^ structures, indicating that the polarity of the C−F bond is weaker than the O−H bond in TFEA.^[^ ^62^, ^63^ ^]^

Figure 6e displays the binding energies of a water molecule (−0.48 eV) and TFEA molecule (−0.69 eV) on the Zn (002) crystal plane, as well as the changes of charge. Obviously, TFEA molecule is more easily adsorbed on the Zn surface, resulting in a larger charge delocalization distribution and an increase in the electrostatic shielding volume. The calculation model consists of a single water molecule or TFEA molecule adsorbed on the Zn (002) surface and a moving Zn^2+^ ion or [Zn−OTf]^+^ contact ion‐pair as shown in Figure 6f–h. The calculated potential energies show an energy barrier of only ≈0.098 eV when a single Zn^2+^ ion passes through a water molecule toward the Zn surface. However, in the presence of a TFEA molecule, the energy barrier is over 1.346 eV, indicating a shielding effect of TFEA on Zn^2+^ ion electromigration. Therefore, a conclusion can be easily derived that in the electrolyte containing TFEA, Zn^2+^ is partially electrostatic shielded by OTf^−^ anions, thereby inhibiting the electromigration of Zn^2+^ near dendrites. At the same time, TFEA can effectively adsorb on the Zn dendrites caused by the tip effect, further increasing the difficulty of Zn^2+^ deposition on dendrites.

**Figure 7:**
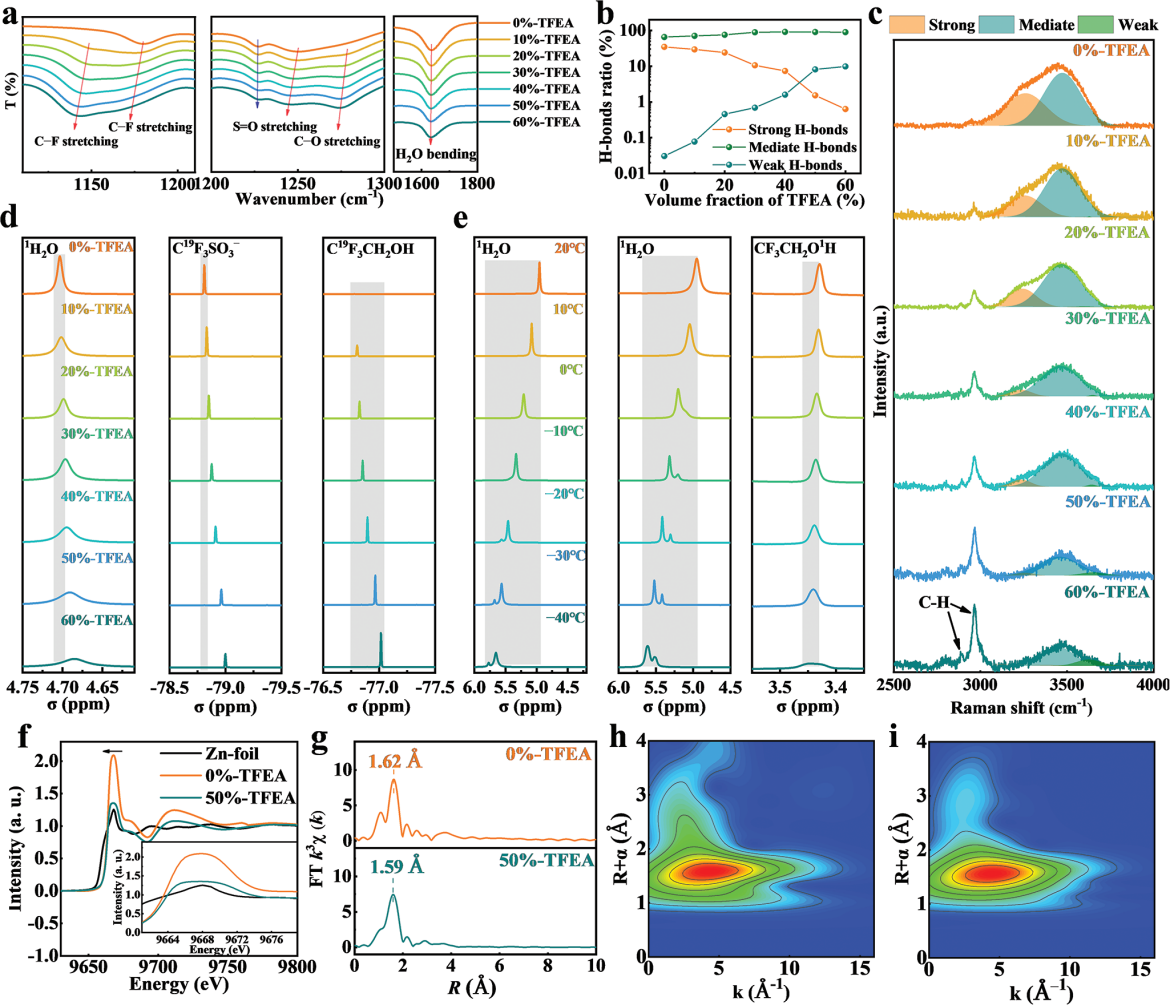
a) Fourier‐transform infra‐red (FTIR) spectroscopy, b) Ratios of strong, mediate, and weak H‐bonds c) Raman spectrum of electrolytes with different volume fraction TFEA. d) 1H and 19F nuclear magnetic resonance (NMR) spectra of the electrolytes with different content of TFEA and e) temperature‐dependent 1H NMR resonance of 0%‐TFEA and 50%‐TFEA. f) Normalized X‐ray absorption near‐edge structure (XANES) spectra, g) extended X‐ray absorption fine structure (EXAFS) spectra in R space, and h,i) wavelet transform images of 0%‐TFEA and 50%‐TFEA.

To further probe the structure changes between solvent molecules and ions in electrolytes, FTIR spectroscopy was conducted as shown in **Figure** **7**a. The C−F stretching vibration peak (1170–1180 cm^−1^) belongs to OTf^−^, undergoing a redshift gradually with an increasing volume fraction of TFEA. Moreover, the asymmetric stretching vibration peaks of S═O (1210–1250 cm^−1^) relating to OTf^−^ close to each other, indicating the formation of [Zn−OTf]^+^ contact ion‐pairs caused by the regulation effect of TFEA. Meanwhile, the peaks at 1130–1140 and 1270–1280 cm^−1^ caused by C−F and C−O stretching vibration in TFEA, as well as the peak (1630–1640 cm^−1^) representing H_2_O bending vibration, also redshift apparently, suggesting that the reshaping coordination structure near water and TFEA molecules. The strength of the H‐bonds among water molecules (strong H‐bonds, medium H‐bonds, weak H‐bonds) can be linked to the extent of disruption in the H‐bonds network by the co‐solvent, which can reflect the difficulty of freezing in the electrolyte at low temperatures.^[^ ^64^, ^65^ ^]^ In general, the stronger H‐bonds between water molecules, the easier to turn into ice at low temperatures.^[^ ^66^, ^67^ ^]^ Figure 7b shows the ratio of the three H‐bonds types at different volume fractions of TFEA (fitted by 3000–3800 cm^−1^ data in the Raman spectrum in Figure 7c). As the content of TFEA increases, the ratio of strong H‐bonds gradually decreases, while the weak H‐bonds exhibit a sharp increase, meaning that TFEA molecules can isolate the water molecules, thus making it difficult to form ice. Meanwhile, the gradual increase in the peak intensity ratio of the C−H bond at 2800–3000 cm^−1^ can be attributed to the larger fraction of TFEA molecules, meaning self‐consistent data.

NMR spectra were performed to investigate the magnetic environment changes of 1H atom and 19F atom. The 1H NMR spectra of 0%‐TFEA exhibit a peak at 4.703 ppm H atm in H_2_O, and then it shifts to 4.685 after adding 60% volume fraction TFEA (Figure 7d). Meanwhile, the 1H resonances from CH_2_ of TFEA at 3.874–3.920 ppm also exhibit a shift toward a lower field, suggesting the potential involvement of TFEA in disrupting the H‐bonds network between water molecules, resulting in an alteration in the magnetic environment of the H atom (Figure S20a, Supporting Information). The 19F NMR spectra show two peaks at −78.809 and −76.804 ppm, which is attributed to the F atom in OTf^−^ and TFEA, respectively. Those peaks have a lower shift to −78.999 ppm and −77.015 at the same time, demonstrating the production of [Zn−OTf]^+^ contact ion‐pairs. To further verify the destructive effect of TFEA on the H‐bonds network in water at low temperatures, deuterated solvent CD_3_OD, serving as an antifreeze, was employed to dissolve 0%‐TFEA and 50%‐TFEA as shown in Figure 7e. Significantly, with the decreasing of the temperature, the original peak gradually split into two peaks, mainly because H_2_O and HDO exist in different proportions in the two electrolytes. Moreover, two higher shifts of 0%‐TFEA (0.748 ppm) and 50%‐TFEA (0.613 ppm) electrolytes can be observed during the decreasing of test temperature. Obviously, the suppressed shift in modified electrolyte exhibits that the freezing process of water at low temperatures is disrupted by TFEA, resulting in a lowered freezing point of the electrolyte, which is consistent with the results of DSC (Figure 1e). Similarly, those peaks representing CH_2_ also experience a corresponding shift (Figure S20b, Supporting Information), reiterating the regulatory role of TFEA on the solvent structure inside the electrolyte.

The X‐ray absorption fine structure ‐ analysis, as a precise tool, was also conducted to verify the solvation structure of Zn^2+^. Compared to 0%‐TFEA characterized by XANES spectrum, 50%‐TFEA exhibits significant changes in both the intensity and shape of the peak at 9668 eV, accompanied by a slight decrease in energy, demonstrating that Zn^2+^ has different solvation structures (Figure 7f). According to the above discussion of molecular dynamics simulations, OTf^−^ can provide more electrons to Zn^2+^ in [Zn(H_2_O)_5_OTf]^+^ solvation structure, leading to a decrease in valence of solvation structure (Figure 6b–d). The Fourier transformation of EXAFS spectra of 0%‐TFEA and 50%‐TFEA exhibit two strongest peaks at 1.62 Å and 1.59 Å in R space, respectively (Figure 7g). This explains the electrostatic attraction effect between OTf^−^ and Zn^2+^ in [Zn(H_2_O)_5_OTf]^+^ structure, resulting in a closer coordination distance between the two ions. Figure 7h and i reveals the differences in the changes of coordination environment of Zn^2+^ by more audio‐visually. The agreement between theoretical simulation calculations and experimental data strongly validates that the optimization effect of TFEA on Zn^2+^ solvation structure, thereby further improving the deposition behavior of Zn^2+^.

The wettability test of V_2_O_5_ cathode is displayed in Video S1 (Supporting Information). When 0%‐TFEA is dropped onto the surface of the V_2_O_5_ cathode, a large droplet forms on the cathode, indicating the poor wettability of 0%‐TFEA. On the contrary, the first droplet of 50%‐TFEA wets the surface of the V_2_O_5_ cathode completely. Moreover, 0%‐TFEA gradually turns yellow after soaking the V_2_O_5_ cathode, while there is almost no color change in 50%‐TFEA after soaking, indicating the enhanced stability of V_2_O_5_ in 50%‐TFEA (Figure S21, Supporting Information). As shown in Figure 1d, the pH value of 0%‐TFEA is 4.15, meaning that the most stable V‐species is VO_2_(OH)_2_^−^ by the phase diagram of V_2_O_5_ and H_2_O system.^[^ ^61^, ^68^ ^]^ In this case, the V_2_O_5_ dissolution mechanism in 0%‐TFEA can be concluded as follows:

(1) $$V_{2}O_{5}+3H_{2}O=2VO_{2}{{\left(\mathrm{OH}\right)}_{2}}^{-}+2H^{+}$$

Furthermore, the DFT calculation result demonstrates the adsorption energies of a TFEA molecule around unsaturated V or O atoms in V_2_O_5_ are much lower than that of a H_2_O molecule, supporting that TFEA molecule can isolate H_2_O and V_2_O_5_, thereby inhibiting the dissolution of V_2_O_5_ in Figure S22 (Supporting Information).^[^ ^69^ ^]^ Even during the repetitive insertion/extraction of Zn^2+^, the V_2_O_5_ cathode still maintains excellent stability in the electrolyte containing TFEA (Figure S23, Supporting Information). The inductively coupled plasma data shows that the concentrations of V element after soaking and charge–discharge times in 50%‐TFEA are always much lower than that in 0%‐TFEA, further proving that the positive effect of TFEA on V_2_O_5_ (Figure S24a,b, Supporting Information). As shown in Figure S24c (Supporting Information), V_2_O_5_ cathode can dissolve in 0%‐TFEA and produce H^+^, thus decreasing the pH of 0%‐TFEA. Meanwhile, the pH values of the electrolytes after different charge‐discharge cycles are shown in Figure S24d (Supporting Information). The increase in pH values once again confirms the irreversible embedding of H^+^ into the V_2_O_5_ lattice, thereby improving the structural stability of V_2_O_5_. Therefore, the above results indicate that TFEA can not only increase the wettability of V_2_O_5_ cathode, but also inhibit the dissolution reaction of V_2_O_5_ by H_2_O, thereby improving the electrochemical performance of the cathode.

In addition to the characterization of the morphology and composition of the electrolytes after reacting with the cathode, more convincing electrochemical tests were conducted to gain a deeper understanding of the positive effects of TFEA on V_2_O_5_. Galvanostatic intermittent titration technique measurement reveals that the electrolyte with TFEA enables the V_2_O_5_ cathode to achieve higher diffusion kinetics with an average ion diffusion coefficient of 3.29 × 10^−12^ cm^2^ s^−1^ at initial discharging due to its good wettability (Figure S25, Supporting Information). Figure S26 (Supporting Information) shows the differential capacity curves of the full batteries during the initial activation stage. Compared with 0%‐TFEA, the curve of 50%‐TFEA at the first cycle shows a significant reduction peak at 0.84 V, indicating the embedding of H^+^.^[^ ^70^ ^]^ With the consumption of H^+^, the differential capacitance curves of 50%‐TFEA exhibit two more stable pairs of redox peaks (1.15 V, 0.96 V and 0.78 V, 0.48 V), implying that the insertion/extraction of Zn^2+^ is the dominant energy storage mechanism in the following cycle. Moreover, the discharge capacity of 50%‐TFEA exhibits the highest capacity of 61.86 mA h g^−1^ at the 1st cycle and suddenly decreases at the 2nd cycle (Figure S27, Supporting Information). Moreover, the first charge capacity is only 35.29 mA h g^−1^, meaning that the H^+^ ions have not completely extracted from V_2_O_5_. These H atoms remaining can stabilize the layered structure of V_2_O_5_, thereby avoiding collapse during the repeated charge‐discharge process.^[^ ^70^, ^71^ ^]^ Subsequently, the capacity presents a gradual increase resembling the trend of 0%‐TFEA, which once again confirms that the energy storage mechanism is progressively dominated by the insertion/extraction of Zn^2+^ during the following cycles.

**Figure 8:**
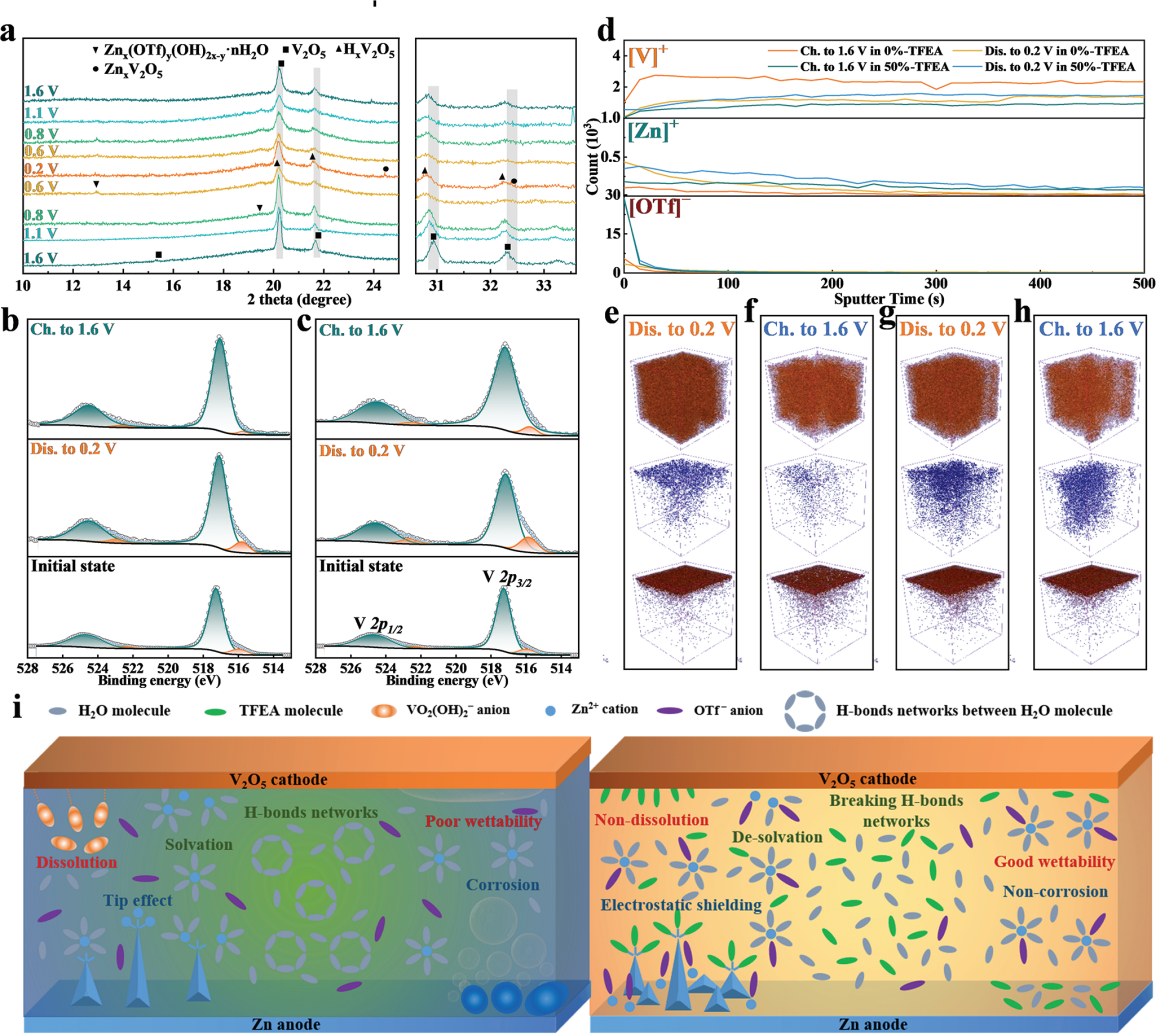
a) Ex situ XRD patterns of V_2_O_5_ cathode in 50%‐TFEA. X‐ray photoelectron spectroscopy of V *2p* of V_2_O_5_ cathode in b) 0%‐TFEA and c) 50%‐TFEA. d) Variation of [Zn]^+^, [V]^+^, and [OTf]^−^ fragment signals with etching time by positive mode or negative mode. 3D variation of signal intensity of various charged fragments of cathodes in 0%‐TFEA e,f) and 50%‐TFEA g,h). i) Schematic illustrations of the effects of 0%‐TFEA and 50%‐TFEA in full batteries.

To verify the phase transition process of the cathode during the first cycle, the *ex‐situ* XRD measurement was operated as shown in **Figure** **8**a and Figure S28 (Supporting Information). The diffraction peaks at 20.3° and 21.8° corresponding to the (001) and (101) crystal planes clearly move to lower angles during the first discharge process, implying that the insertion of H^+^ ion leads to an increase of the crystal plane spacing. More importantly, some new peaks located at 12.9° and 19.4° corresponding to Zn_x_(OTf)_y_(OH)_2x−y_·nH_2_O emerge during the first discharging process, suggesting the insertion of H^+^ ions.^[^ ^72^ ^]^ These new peaks gradually weaken during the charging process, indicating the extraction of partly H^+^ ions. The peaks located at 24.5° and 32.4° belong to Zn_x_V_2_O_5_ at 0.2 V. In the V *2p* spectrum of 0%‐TFEA, two obvious characteristic peaks of V^4+^ (515.8 and 522.5 eV) appear at 0.2 V, while those peaks gradually disappear when charging up to 1.6 V, indicating that the V atoms containing unsaturated bonds are dissolved by water at this voltage (Figure 8b).^[^ ^70^, ^71^, ^72^, ^73^ ^]^ Fortunately, the V_2_O_5_ cathode still contains a certain amount of V^4+^ in 50%‐TFEA due to the insertion of H^+^ and the inhibition effect of V_2_O_5_ dissolution reaction by TFEA (Figure 8c). Figure S29 (Supporting Information) shows that the Zn element also retains on the surface of cathodes due to the presence of Zn_x_(OTf)_y_(OH)_2x−y_·nH_2_O. The results above are consistent with the differential capacitance curve (Figure S26, Supporting Information).

To visually display the distribution of various components on the surface of the V_2_O_5_ cathode, time of flight secondary ion mass spectrometry (TOF‐SIMS) measurements were conducted as shown in Figure S30 (Supporting Information). After soaking in 0%‐TFEA for 1 week, a large number of fragments [CF_3_]^+^ belonging to the binder (Polyvinylidene fluoride) are detected on the surface of the cathode due to the dissolution of V_2_O_5_, which obviously hinders the diffusion of Zn^2+^. However, this phenomenon is suppressed in 50%‐TFEA apparently, indicating that TFEA can enhance the stability of V_2_O_5_. Figure 8d reveals the variation of [Zn]^+^, [V]^+^, and [OTf]^−^ fragment signals with etching time. Unlike the [Zn]^+^ fragment, the [OTf]^−^ fragment sharply decreases, indicating that Zn_x_(OTf)_y_(OH)_2x−y_·nH_2_O is mainly distributed at the interface between cathode and electrolyte. Figure 8e–h show the 3D variation of the TOF‐SIMS intensity related to the corresponding charged fragments. Compared with 0%‐TFEA, whether discharging to 0.2 V or charging to 1.6 V, more Zn^2+^ and OTf^−^ appear at the depth of the cathode mainly because of the excellent wettability of 50%‐TFEA.^[^ ^74^, ^75^ ^]^ To sum up, the electrochemical performance of V_2_O_5_ cathode determines the electrochemical performance of the full battery. This is mainly because Zn anode can provide more Zn^2+^, but the ability to store those Zn^2+^ from anode is not enough. In fact, TEFA can improve the structural stability and wettability of V_2_O_5_ cathode in electrolyte. Due to the adsorption effect of TFEA, the dissolution of V_2_O_5_ in the electrolyte is effectively prevented, thus inhibiting the loss of V_2_O_5_ during the working process. On the other hand, the insertion of H^+^ can also stabilize the structural stability of V_2_O_5_. Based on those positive effects, the utilization rate of the cathode material increases, thereby improving the cycle life and capacity.

The schematic illustrations of the effects of the two electrolytes are shown in Figure 8i. The positive effects of TFEA described earlier can be summarized as follows: Inhibiting dendrites and corrosion for Zn anodes; Disrupting the H‐bonds networks between water molecules and promoting de‐solvation of Zn^2+^ for electrolytes; Avoiding the dissolution and promoting wettability for V_2_O_5_ cathode. This implies that TFEA has “shooting three birds with one stone” effect on full batteries, and highlights the promise of a successful electrolyte regulation strategy for the development of high‐performance and practical ZIBs.

## 3 Conclusion

In summary, we have successfully found a polarity soluble organic solvent that has a triple‐function on the anode, electrolyte, and cathode simultaneously, enabling highly reversible and performance ZIBs. TFEA promotes the formation of the [Zn(H_2_O)_5_(OTf)]^+^ solvation structure and shields the negative charge of Zn dendrites, thus promoting the uniform deposition on the Zn anode. Meanwhile, TFEA can also destroy the H‐bonds network in water, causing a decline in the freezing point of the electrolyte, thereby improving its low‐temperature performance. Furthermore, TFEA can inhibit the dissolution of V_2_O_5_, accelerate the electrolyte wettability in V_2_O_5_, and H^+^ ion diffusion, thereby can ensure the reliability of V_2_O_5_ cathode. Therefore, based on the gain effects from TFEA mentioned above, the V_2_O_5_//Zn full batteries with TFEA exhibit the capacities of 272.44 and 116.78 mA h g^−1^ at room and low temperatures, respectively. Under the condition of only 1.3 M Zn(OTf)_2_, using low‐concentration electrolytes directly paves a significant step toward commercialized ZIBs to break the low‐temperature capacity limit. Our work provides an exact view of verifying the compatibility of electrolyte co‐solvents, which improves the validation standards for electrolyte co‐solvents and boosts the development of the research on ZIBs.

## Conflict of Interest

The authors declare no conflict of interest.

## Supporting information

Supporting Information

Supplemental Video 1

## Data Availability

The data that support the findings of this study are available from the corresponding author upon reasonable request.
