# Supplementary material for: A Stable High‐Performance Zn‐Ion Batteries Enabled by Highly Compatible Polar Co‐Solvent
Source: Adv Sci (Weinh). 2024 Jul 17;11(35):2403513. doi: 10.1002/advs.202403513 (PMC11425257; doi:10.1002/advs.202403513)
Supplement: Supplementary file 1 — Supporting Information [file ADVS-11-2403513-s002.docx]

**Supporting Information**

**A Stable High-Performance Zn-ion Batteries Enabled by Highly Compatible Polar Co-solvent**

Shuo Yang ^a, b^, Guangpeng Wu ^a^, Jing Zhang ^a^, Yuning Guo ^a^, Kui Xue ^a^, Yongqi Zhang ^b^, Yuanmin Zhu ^c^, Tao Li ^d*^, Xiaofeng Zhang ^a*^, Liujiang Zhou ^a*^

a: School of Physics, State Key Laboratory of Electronic Thin Films and Integrated Devices, University of Electronic Science and Technology of China, Chengdu, 611731 China.

b: Institute of Fundamental and Frontier Sciences, University of Electronic Science and Technology of China, Chengdu, 611731, China.

c: Research Institute of Interdisciplinary Science & School of Materials Science and Engineering, Dongguan University of Technology, Dongguan, 523808, China.

d: Institute of Materials and Physics, Ganjiang Innovations Academy, Chinese Academy of Sciences, Ganzhou, 341119, China.

E-mail addresses:

ljzhou@uestc.edu.cn,

xfzhang_2022@uestc.edu.cn,

litao@gia.cas.cn.

**1. Experimental**

**1.1. Materials**

Vanadium pentoxide (V_2_O_5_), Zinc trifluoromethanesulfonate (Zn(OTf)_2_, AR), and trifluoroethanol (TFEA, AR) were purchased from Macklin (Shanghai, China). Stainless steel mesh, carbon paper, and conductive carbon black were purchased from Saibo Electrochemical Materials Network. 0.1 mm high-purity zinc foil, 0.02 mm copper foil and glass fiber separators (GF/B) were also purchased online. 2032 type button cells were purchased from NEWARE.

**1.2. Synthesis of electrolyte and V_2_O_5_ cathode**

All electrolytes were prepared by the concentration dilution method. First, we weighed 23.63 g of Zn(OTf)_2_ in a 50 mL volumetric flask. Next, we added deionized water to the flask to 20 mL and then stirred the solution for 72 h at 40℃. The obtained solution was diluted to 50 ml by deionized water to obtain the 1.3 M Zn(OTf)_2_ electrolyte (0%-TFEA). Similarly, TFEA was used instead of deionized water to reach 50 mL, named as X%-TFEA (X = the volume fraction of TFEA). All electrolytes were freshly prepared. 0.7 g V_2_O_5_ and 0.2 g carbon black (Super P) were weighed in a mortar, and ground for 30 min. Subsequently, the N-Methylpyrrolidone (NMP) solution containing 0.1 g polyvinylidene fluoride (PVDF) were added in mortar. After thorough mixing, the synthesized mixture was applied onto the stainless-steel mesh and dried at 60℃ for 24 h. The loading mass of V_2_O_5_ is approximately ~2 mg cm^−2^.

**1.3. Characterization**

The morphology, phases, and chemical states of the Zn surface after stripping/plating cycles were comprehensively characterized using advanced analytical techniques including a scanning electron microscope (SEM; ZEISS Sigma 300), laser scanning confocal microscopy (LSCM; KEYENCE VK-X150), and X-ray diffraction (XRD; Rigaku Ultima IV with Cu-Kα radiation). The viscosities and ionic conductivities of the electrolytes were precisely measured using Antonpa Lovis 2000 M and DDS 307, respectively. The temperature of the phase-transition reaction of the electrolyte was investigated using a differential scanning calorimeter (DSC; Netzsch DSC 200 F3). To verify the changes in bonds within the electrolyte, Raman spectroscopy (LabRAM HR Evolution) and Fourier transform infrared spectroscopy (FITR) in attenuated total reflectance (ATR) mode from Bruker ALPHA II (Germany) were employed to detect electrolyte. Additionally, Nuclear Magnetic Resonance spectroscopy (NMR) analysis of 1H and 19F was performed on a Bruker Avance NEO 600MHz (Germany) to further illustrate the coordination bond changes in various electrolytes. The pH value of the electrolyte was also precisely measured using a pH measurement (FE-28Standard). X-ray photoelectron spectroscopy (XPS) was taken from a Thermo Scientific K-Alpha spectrometer to conduct for elemental determination. Ionic concentrations in electrolytes were analyzed by inductively coupled plasma atomic emission spectrometry (ICP). *In-situ* optical microscopy was used to observe the Zn anode during the Zn plating process in different electrolytes at 1 mA cm^−1^. Time of flight secondary ion mass spectrometry (TOF-SIMS) measurements were conducted with a PHI nano TOF 3. A Bi_3_^++^ beam (30 keV, 2 nA, 5 × 5 μm^2^) was used as the primary beam to detect the samples, sputtering with an Ar^+^ beam (1 keV, 100 nA, 400 × 400 μm^2^) was applied for depth profiling analysis. Zn K-edge X-ray absorption fine structure (XAFS) spectra analyses were performed with Si (111) crystal monochromators at the BL14W Beam line at the Shanghai Synchrotron Radiation Facility (Shanghai, China). Before the analysis at the beamline, samples were placed into aluminum sample holders and sealed using Kapton tape film. The XAFS spectra were recorded at room temperature using a 4-channel Silicon Drift Detector Bruker 5040. Zn K-edge extended X-ray absorption fine structure (EXAFS) spectra were recorded in transmission mode. The XAFS spectra of these standard samples were recorded in transmission mode. The obtained XAFS data was processed in Athena for background,^1^ pre-edge line and post-edge line calibrations. Then Fourier transformed fitting was carried out in Artemis.^2^ The k^3^ weighting, k-range of 3~12 Å^−1^ and R range of 1~3 Å were used for the fitting of zinc foil; The k^3^ weighting, k-range of 3~11 Å^−1^ and R range of 1~2 Å were used for the fitting of electrolytes. For Wavelet Transform analysis, the χ(k) exported from Athena was imported into the Hama Fortran code. The parameters were listed as follows: R range, 0~4 Å; k range, 0~16 Å^−1^; k weight, 2; and Morlet function with κ=10, σ=1 was used as the mother wavelet to provide the overall distribution. The *ex-situ* XRD measurement was operated by using a home-made electrochemical button cell with an 8 mm diameter window. The scanning speed is 5°/min, with a range of 10~50°.

**1.4. Electrochemical measurements**

Cyclic voltammetry (CV) and Tafel curves with different electrolytes were measured in a three-electrode system. For the CV test, copper foil, Zn foil and saturated calomel electrode (SCE) were used as the working electrode, the counter electrode and the reference electrode, respectively. For Tafel polarization tests, both the working electrode and counter electrode were zinc foil, and SCE was the reference electrode. The scanning rates of CV and Tafel curves were 10 mV s^−1^. Electrochemical impedance spectroscopy (EIS) tests were carried out at a frequency range of 100000~0.01Hz under the over-voltage for Zn//Zn symmetry cells or open-circuit voltage for V_2_O_5_ full batteries. The working voltage range of the electrolyte was tested with stainless steel sheet (Working electrode and counter electrode) and SCE (Reference electrode) by linear sweep voltammetry (LSV) at 10 mV s^−1^. Cu//Zn half cells and Zn//Zn symmetry cells were assembled with GF/B separator (*φ* = 1.9 cm), copper foil (*φ* = 1.6 cm) and zinc foil (*φ* = 1.6 cm) under 8 MPa pressure. For full batteries, V_2_O_5_ were used as cathode materials, the current collector was stainless steel mesh (*φ* = 1.2 cm) and zinc foil (*φ* = 1.2 cm) was used as the anode. Galvanostatic curves and cycle performances were tested by the Neware battery instrument at 25℃ or −25℃. The low-temperature test of full batteries was carried out in a thermal test chamber (MGDW-GWX-250L). Before cycling, the V_2_O_5_//Zn full batteries should be quieting for 24 h after assembling and activated for 48 h at a current density of 0.1 A g^−1^. Besides, the full batteries did not be treated in any way to gain accurate differential capacitance and galvanostatic intermittent titration technology (GITT) curves at the beginning of operation. All electrochemistry performance tests and cycling performance tests were conducted using the NEWARE instrument and Chenhua CHI760e electrochemical workstation.

**1.5. Computer simulation**

**1.5.1 Molecular dynamics simulations**

**MD simulations.** Molecular dynamics (MD) simulations for the solvation structure of aqueous electrolytes were conducted by using the LAMMPS package. The forced field parameters for Zn^2+^, OTf^-^, and trifluoroethanol were obtained from OPLS_AA force fields. The molecular ratio of numbers of Zn(OTf)_2_, H_2_O and TFEA is 1.9 : 98.1 : 0 (0%-TFEA) and 3.2 : 74.4 : 22.4 (50%-TFEA). The SPC/E water model was employed for H_2_O. The bonds and the angle of water molecules were constrained with SHAKE algorithm. The velocity-verlet method was used as an integral method and the integration time step was 1 fs. A cutoff radius of 15 Å was applied in the MD simulations of vdW and electrostatic interactions. The standard periodic boundary condition was used in all simulations. Particle-particle particle-mesh (PPPM) solver was applied to describe the long electrostatic interactions. First, the energy minimization was performed to obtain a stable system. After minimization, each system was then equilibrated under the NPT ensemble at a constant temperature of 293.15 K for 5 ns. Finally, each system was simulated for the last 1000 ps under NVT ensemble for data collection. Nosé–Hoover thermostat/barostat algorithm was used to control the temperature and pressure of each system in the NPT and NVT ensemble. The snapshot of the MD simulation is produced by OVITO software.

**1.5.2 DFT calculations.** All calculations were performed by using the projector augmented wave (PAW) method within density functional theory (DFT) as implemented in the Vienna *ab initio* Simulation Package (VASP).^3,4,5^ Generalized gradient approximation (GGA) parameterized by Perdew-Burke-Ernzerhof (PBE) formula was employed for evaluating the electron exchange correlation energy.^6^ The DFT-D3 method of Grimme was used to describe the weak dispersion forces.^7^ A cutoff energy of 400 eV and a *Г*-centered 1×1×1 *k*-point grids were adopted. The structural parameters and all the atoms were fully optimized until the Hellman-Feynman forces were less than 0.02 eV/Å. The binding energies of AB solvated molecules were obtained by $E_{b}=E\left( AB \right)-\left[ E\left( A \right)+E\left( B \right) \right],$where *E* (AB), *E*(A) and *E*(B) are the ground-state energy of AB molecule, A molecule, and B molecule, respectively. A solvated molecule was in a periodic cubic box with a length of 20 Å to minimize the interaction between periodic images. The adsorption energy (*E*_ad_) can be described as$E_{ad}=E\left( sub \right)+E\left( sm \right)-E(sub+sm)$, where *E*_sub_ and *E*(sub + sm) represent the energies of substrates without and with the adsorbed solvent molecules (H_2_O/Trifluoroethanol), respectively, and *E*(sm) is the energy of the isolated solvent molecules. The charge density difference was calculated as $\Delta\rho=\rho\left( sub+sm \right)-(\rho\left( \mathrm{sub} \right)+\rho(sm))$, where ρ(sub) and ρ(sub + sm) correspond to the charge density of substrates without and with the adsorbed molecules (H_2_O/Trifluoroethanol), respectively, and ρ(sm) is the charge density of the isolated solvent molecules. The Zn (001) surface is modeled by a five-layer 5 × 5 surface supercell and a vacuum layer thickness of 20 Å suited to all the slab models.

**Table S1**. Comparison of price, specification, and purity of various monohydric alcohols containing F atoms.

| Species | Price (USD g^−1^) | Specification (g) | Purity (%) |
| --- | --- | --- | --- |
| 2-fluoroethanol | 1.39 | 100 | 95 |
| 2,2-difluoroethanol | 0.11 | 500 | 97 |
| TFEA | 0.07 | 5000 | 99.5 |
| 1,1,1-trifluoro-2-propanol | 5.55 | 100 | 97 |
| 2,2,3,3,3-pentafluoropropanol | 0.71 | 100 | 98 |
| 1,1,1,3,3,3-hexafluoroisopropanol | 0.15 | 2500 | 99.5 |
| 4,4,4-trifluorobutanol | 1.67 | 100 | 98 |
| 2,2,3,3,4,4,4-heptafluorobutanol | 2.71 | 100 | 98 |

**Table S2**. Comparison of current density, areal capacity, and cycle life of Zn//Zn symmetric cells with various electrolytes reported in references.

| Electrolyte | Current density (mA cm^−2^) | Areal capacity (mA h cm^−2^) | Cycle life (h) | Reference |
| --- | --- | --- | --- | --- |
| 1.3 M Zn(OTf)_2_ | 5 | 2 | 782 | **This work** |
| 30 M ZnCl_2_ | 0.2 | 0.2 | 4300 | [8] |
| 1 M ZnSO_4_ | 1 | 0.5 | 1100 | [9] |
| 2 M ZnSO_4_ | 2 | 2 | 2000 | [10] |
| 2 M ZnSO_4_ | 5 | 5 | 460 | [11] |
| 2 M ZnSO_4_ | 1 | 1 | 550 | [12] |
| 2 M ZnSO_4_ | 2 | 1 | 2400 | [13] |
| 2 M ZnSO_4_ | 5 | 1 | 1600 | [14] |
| 3 M ZnSO_4_ | 2 | 2 | 800 | [15] |
| ~3 M ZnSO_4_ | 0.2 | 0.2 | 3000 | [16] |
| 1 M Zn(OTf)_2_ | 1 | 1 | 980 | [17] |
| 2 M Zn(OTf)_2_ | 1 | 1 | 1000 | [18] |

**Table S3**. Comparison of current density and specific capacity of full batteries with Zn(OTf)_2_ electrolyte and V-based cathode reported in references.

| Electrolyte | Cathode Materials | Current density  (A g^−1^) | Specific capacity  (mA h g^−1^) | Reference |
| --- | --- | --- | --- | --- |
| 1.3 M Zn(OTf)_2_ | V_2_O_5_ | 2 | 272 | **This work** |
| 0.5 M Zn(OTf)_2_ | V_2_O_5_ | 0.5 | ~160 | [19] |
| 1 M Zn(OTf)_2_ | V_2_O_5_ | 10 | ~130 | [20] |
| 2 M Zn(OTf)_2_ | V_2_O_5_·nH_2_O | 2 | ~260 | [21] |
| 2 M Zn(OTf)_2_ | VO_2_ | 1 | 288.8 | [22] |
| 2 M Zn(OTf)_2_ | NH_4_V_4_O_10_ | 0.5 | 420 | [23] |
| 3 M Zn(OTf)_2_ | V_2_O_5_ | 5 | ~400 | [24] |
| 3 M Zn(OTf)_2_ | V_2_O_5_·nH_2_O | 6 | 228 | [25] |
| 3 M Zn(OTf)_2_ | Na_2_V_6_O_16_·1.63H_2_O | 0.1 | 296 | [26] |

**Table S4.** Comparison of operation temperature, current density, cycle number, and specific capacity of full batteries with various electrolytes reported in references.

| Electrolyte | Operation temperature (℃) | Current density (A g^−1^) | Cycle number | Specific capacity (mA h g^−1^) | Reference |
| --- | --- | --- | --- | --- | --- |
| 1.3 M Zn(OTf)_2_, 50% TFEA, | 25 | 2 | 1000 | 247 | **This work** |
| 1.3 M Zn(OTf)_2_, 50% TFEA, | −25 | 2 | 2000 | 102 | **This work** |
| 1 M ZnCl_2_, 10% TG, | 25 | 4 | 1000 | 124 | [27] |
| 1 M ZnSO_4_, 10 mM glucose | 25 | 3.08 | 1000 | 112 | [28] |
| 1 M Zn(OTf)_2_, 56% ME | 25 | 10 | 1500 | 152 | [29] |
| 1 M Zn(OTf)_2_, 50% PC | −20 | 0.2 | 200 | 78 | [30] |
| 1 M ZnSO_4_, 4 EMImCl | 25 | 1 | 300 | 160 | [31] |
| 1 M Zn(OTf)_2_, 50% DES | 25 | 0.5 | 500 | 114 | [32] |
| 1 M ZnSO_4_, 1% Butanediol | 40 | 0.4 | 250 | 142 | [33] |
| 2 M ZnSO_4_, 50% ME | −10 | 5 | 2000 | 115 | [34] |
| 2 M ZnSO_4_, 1 M Z10 | 25 | 1 | 1000 | 201 | [35] |
| 2 M Zn(OTf)_2_, 0.1 M MSG | 25 | 2 | 1000 | 183 | [36] |
| 2 M ZnSO_4_, 2 mM TA | 25 | 1 | 1000 | 182 | [37] |
| 2 M ZnSO_4_, 50 mM SL | 25 | 3 | 500 | 242 | [38] |
| 2 M ZnSO_4_, 0.5 g L^−1^ SN | 25 | 20 | 3350 | 87 | [39] |
| 2 M ZnSO_4_, 0.2 M MnSO_4_, 30 Mm LiODFB | 25 | 2 | 2000 | 136 | [40] |
| 3 M ZnSO_4_, 10 mM α-CD | 25 | 3 | 800 | 280 | [41] |
| 3 M Zn(OTf)_2_, 1% PAA | −25 | 1 | 324 | 227 | [42] |
| 4 M Zn(OTf)_2_, 20% TEP | 25 | 0.1 | 210 | 317 | [43] |

**Table S5**. Numbers of various H-bonds in the different electrolytes in MD models.

| Electrolytes  H-bonds type | 0%-TFEA | 50%-TFEA |
| --- | --- | --- |
| O(H_2_O)−H(H_2_O) | 1775 | 605 |
| O(OTf^−^)−H(H_2_O) | 157 | 124 |
| O(TFEA)−H(H_2_O) | 0 | 102 |

**Reference**

[1] Funke, H.; Scheinost, A. C.; Chukalina, M. Wavelet analysis of extended X-ray absorption fine structure data. *Physical Review*, B ***2005*** *71*, 094110.

[2] Funke, H.; Chukalina, M.; Scheinost, A. C. A new FEFF-based wavelet for EXAFS data analysis. *Journal of Synchrotron Radiation* ***2007***, *14*, 426-432.

[3] Kresse, G.; Joubert, D. From ultrasoft pseudopotentials to the projector augmented-wave method. *Physical Review B* ***1999***, *59* (3), 1758-1775.

[4] Kresse, G.; Furthmuller, J. Efficient iterative schemes for ab initio total-energy calculations using a plane-wave basis set. *Physical Review B* ***1996***, *54* (16), 11169-11186.

[5] Kresse, G.; Furthmuller, J. Efficiency of ab-initio total energy calculations for metals and semiconductors using a plane-wave basis set. *Computational Materials Science* ***1996*,** *6* (1), 15-50.

[6] Perdew, J. P.; Burke, K.; Ernzerhof, M. Generalized gradient approximation made simple. *Physical Review Letters* ***1996***, *77* (18), 3865-3868.

[7] Grimme, S.; Antony, J.; Ehrlich, H. S. Krieg, A consistent and accurate ab initio parametrization of density functional dispersion correction (DFT-D) for the 94 elements H-Pu. *Journal of Chemical Physics* ***2010***, *132* (15) 154104.

[8] Wang R.; Yao M.-J.; Yang M.; Zhu J.-C.; Chen J.; Niu Z.-Q. Synergetic modulation on ionic association and solvation structure by electron-withdrawing effect for aqueous zinc- ion batteries. *Proc. Natl. Acad. Sci*. ***2023***, 120, 2221980120.

[9] Liu, Z.-X.; Wang, R.; Ma, Q.-W.; Wan, J.-D.; Zhang, S.-L.; Zhang, L.-H.; Li, H.-B.; Luo, Q.-Q.; Wu, J.; Zhou, T.-F.; Mao, J.-F.; Zhang, L.; Zhang, C.-F.; Guo, Z.-P. A Dual-Functional Organic Electrolyte Additive with Regulating Suitable Overpotential for Building Highly Reversible Aqueous Zinc Ion Batteries. *Adv. Funct. Mater*. ***2023***, 34, 2214538.

[10] Huang C.; Zhao X.; Hao, Y.; Yang, Y.; Qian, Y.; Chang, G.; Zhang, Yan.; Tang, Q.-L.; Hu, A.-P.; Chen, X.-H. Self-Healing SeO_2_ Additives Enable Zinc Metal Reversibility in Aqueous ZnSO_4_ Electrolytes. *Adv. Funct. Mater.* ***2022***, 32, 2112091.

[11] Hu, Q.; Hu, J.-S.; Li, L.; Ran, Q.-W.; Ji, Y.-Y.; Liu, X.-Q.; Zhao, J.-X.; Xu B.-G. In-depth study on the regulation of electrode interface and solvation structure by hydroxyl chemistry. *Energy Stor. Mater.* ***2023***, 54, 374-381.

[12] Li, T.-C.; Lim, Y.-V.; Li, X.-L.; Luo, S.-Z.; Lin, C.-J.; Fang, D.-L.; Xia, S.-W.; Wang, Y.; Yang, H.-Y. A Universal Additive Strategy to Reshape Electrolyte Solvation Structure toward Reversible Zn Storage. *Adv. Energy Mater*. ***2022***, 12, 2103231.

[13] Lin, C.-Y.; Yang, X.-H.; Xiong, P.-X.; Lin, H.; He, L.-J.; Yao, Q.; Wei, M.-D.; Qian, Q.-R.; Chen, Q.-H.; Zeng, L.-X. High-Rate, Large Capacity, and Long Life Dendrite-Free Zn Metal Anode Enabled by Trifunctional Electrolyte Additive with a Wide Temperature Range. *Adv. Sci*. ***2022***, 9, 2201433.

[14] Liu, H.; Xin, Z.-J.; Cao, B.; Xu, Z.-J.; Xu, B.; Zhu, Q.-Z.; Yang, J.-L.; Zhang, B.; Fan, H.-J. Polyhydroxylated Organic Molecular Additives for Durable Aqueous Zinc Battery. *Adv. Funct. Mater*. ***2023***, 34, 2309840.

[15] Wang, K.; Qiu, T.; Lin, Lu.; Liu, X.-X.; Sun, X.-Q. A low fraction electrolyte additive as interface stabilizer for Zn electrode in aqueous batteries. *Energy Stor. Mater*. ***2023***, 54, 366–373.

[16] Wei T.-T.; Ren, Y.-K.; Li, Z.-Q.; Zhan, X.-X.; Ji, D.-H.; Hu, L.-H. Bonding interaction regulation in hydrogel electrolyte enable dendrite-free aqueous zinc-ion batteries from −20 to 60℃. *Chem. Eng. J*. ***2022***, 434, 134646.

[17] Du, H.-H.; Wang, K.; Sun, T.-J.; Shi, J.-Q.; Zhou, X.-Z.; Cai, W.-S.; Tao, Z.-L. Improving zinc anode reversibility by hydrogen bond in hybrid aqueous electrolyte. *Chem. Eng. J*. ***2022***, 427, 131705.

[18] Qiu, M.-J.; Sun, P.; Qin, A.-M.; Cui, G.-F.; Mai, W.-J. Metal-coordination chemistry guiding preferred crystallographic orientation for reversible zinc anode. *Energy Stor. Mater*. ***2022***, 49, 463–470.

[19] Mei, Y.-Y.; Liu, Y.-H.; Xu, W.; Zhang, M.-H.; Dong, Y.-F.; Qiu, J.-S. Suppressing vanadium dissolution in 2D V_2_O_5_/MXene heterostructures via organic/aqueous hybrid electrolyte for stable zinc ion batteries. *Chem. Eng. J*. ***2023***, 452, 139574.

[20] Wu, Y.; Zhang, T.; Chen, L.-N.; Zhu, Z.-H.; Cheng, L.-K.; Gu, S.; Li, Z.-Q.; Tong, Z.-Q.; Li, H.; Li, Y.-F.; Lu, Z.-G.; Zhang, W.-J.; Lee, C.-S. Polymer Chain-Guided Ion Transport in Aqueous Electrolytes of Zn-Ion Batteries. *Adv. Energy Mater*. ***2023***, 13, 2300719.

[21] Ma, G.-Q.; Miao, L.-C.; Dong, Y.; Yuan, W.-T.; Nie, X.-Y.; Di, S.-L.; Wang, Y.-Y.; Wang, L.-B.; Zhang, N. Reshaping the electrolyte structure and interface chemistry for stable aqueous zinc batteries. *Energy Stor. Mater*. ***2022***, 47, 203–210.

[22] Xu, W.-W.; Li, J.-T.; Liao, X.-B.; Zhang, L.; Zhang, X.-M.; Liu, C.-Z.; Amine, K.; Zhao K.-G.; Lu J. Fluoride-Rich, Organic−Inorganic Gradient Interphase Enabled by Sacrificial Solvation Shells for Reversible Zinc Metal Batteries. *J. Am. Chem. Soc.* ***2023***, 145, 22287–22856.

[23] Chen, R.-W.; Li, J.-W.; Du, Z.-J.; Guo, F.; Zhang, W.; Dai, Y.-H.; Zong, W.; Gao, X.; Zhu, J-X.; Zhao, Y.; Wang, X.-H.; He, G. Hydrated Deep Eutectic Electrolyte with Finely-Tuned Solvation Chemistry for High Performance Zinc-Ion Batteries. *Energy Environ. Sci*. ***2023***, 16, 2540–2549.

[24] Zhang, N.; Dong, Y.; Jia, M.-J.; Bian, X.; Wang, Y.-Y.; Qiu, M.-D.; Xu, J.-Z; Liu, Y.-C.; Jiao, L.-F.; Cheng, F.-Y. Rechargeable Aqueous Zn−V2O5Battery with High Energy Density and Long Cycle Life. ACS EnergyLett.2018,3, 1366−1372.

[25] Yan, M.-Y.; He, P.; Chen, Y.; Wang, S.-Y.; Wei, Q.-L.; Zhao, K.-N.; Xu, X.; An, Q.-Y.; Shuang, Y.; Shao, Y.-Y.; Mueller, K.; Mai, L.-Q.; Liu, J.; Yang, J.-H. Water-Lubricated Intercalation inV_2_O_5_·nH_2_O for High-Capacity and High-Rate Aqueous Rechargeable Zinc Batteries. *Adv. Mater*. ***2018***, 30, 1703725.

[26] Hu, P.; Zhu, T.; Wang, X.-P.; Wei, X.-J; Yan, M.-Y; Li, J.-T; Luo, W.; Yang, W.; Zhang, W.-C.; Zhou, L.; Zhou, Z.-Q.; Mai, L.-Q.; Highly Durable Na_2_V_6_O_16_·1.63H_2_O Nanowire Cathode for Aqueous Zinc-Ion Battery. *Nano Lett*. ***2018***, 18, 1758−1763.

[27] Liu, Z.-X.; Wang, R.; Ma, Q.-W.; Wan, J.-D.; Zhang, S.-L.; Zhang, L.-H.; Li, H.-B.; Luo, Q.-Q.; Wu, J.; Zhou, T.-F.; Mao, J.-F.; Zhang, L.; Zhang, C.-F.; Guo, Z.-P. A Dual-Functional Organic Electrolyte Additive with Regulating Suitable Overpotential for Building Highly Reversible Aqueous Zinc Ion Batteries. *Adv. Funct. Mater.* ***2023***, 2214538.

[28] Sun, P.; Ma, L.; Zhou, W.-H.; Qiu, M.-J.; Wang, Z.-L.; Chao, D.-L.; Mai, W.-J. Simultaneous Regulation on Solvation Shell and Electrode Interface for Dendrite-Free Zn Ion Batteries Achieved by a Low-Cost Glucose Additive. *Angew. Chem. Int. Ed.* ***2021***, 60,18247–18255.

[29] Xu, W.-W.; Li, J.-T.; Liao, X.-B.; Zhang, L.; Zhang, X.-M.; Liu, C.-Z.; Khalil, A.; Zhao, Z.-N.; Lu, J. Fluoride-Rich, Organic-Inorganic Gradient Interphase Enabled by Sacrificial Solvation Shells for Reversible Zinc Metal Batteries. *J. Am. Chem. Soc.* ***2023***, 145, 22456−22465.

[30] Ming, F.-W.; Zhu, Y.-P.; Huang, G.; Emwas, A.-H.; Liang, H.-F.; Cui, Y.; Husam N.-A. Co-Solvent Electrolyte Engineering for Stable Anode-Free Zinc Metal Batteries. *J. Am. Chem. Soc.* ***2022***, 144, 7160−7170.

[31] Zhang, Q.; Ma, Y.-L.; Lu, Y.; Zhou, X.-Z.; Lin, L.; Li, L.; Yan, Z.-H.; Zhao, Q.; Zhang, K.; Chen, J. Designing Anion-Type Water-Free Zn^2+^ Solvation Structure for Robust Zn Metal Anode. *Angew. Chem. Int. Ed.* ***2021***, 60, 23357–23364.

[32] Wang, S.-H.; Liu, G.-X.; Wan, W.; Li, X.-Y.; Li, J.; Wang, C. Acetamide-Caprolactam Deep Eutectic Solvent-Based Electrolyte for Stable Zn-Metal Batteries. *Adv. Mater.* ***2023***, 2306546.

[33] Shang, Y.; Kundi, V.; Pal, I.; Kim, H.-N.; Zhong, H.-Y.; Kumar, P.; Kundu, D. Highly Potent and Low-Volume Concentration Additives for Durable Aqueous Zinc Batteries: Machine Learning-Enabled Performance Rationalization. *Adv. Mater.* ***2023***, 2309212.

[34] Hao, J.-N.; Yuan, L.-B.; Ye, C.; Chao, D.-L.; Davey, K.; Guo, Z.-P.; Qiao, S.-Z. Boosting Zinc Electrode Reversibility in Aqueous Electrolytes by Using Low-Cost Antisolvents. *Angew. Chem. Int. Ed.* ***2021***, 60, 7366–7375.

[35] Tao, L.; Guan, K.-L.; Yang, R.; Guo, Z.-X.; Wang, L.-Y.; Xu, L.; Wan, H.-Z.; Zhang, J.; Wang, H.-B.; Hu, L.-F.; Dyson, P.-J.; Nazeeruddin, M.-K.; Wang, H. Dual-protected zinc anodes for long-life aqueous zinc ion battery with bifunctional interface constructed by zwitterionic surfactants. *Energy Stor. Mater.* ***2023****,* 63, 102981.

[36] Zhong, Y.; Cheng, Z.-X.; Zhang, H.-W.; Li, J.-B.; Liu, D.-D.; Liao, Y.-Q.; Meng, J.-T.; Shen, Y.; Huang, Y. Monosodium glutamate, an effective electrolyte additive to enhance cycling performance of Zn anode in aqueous battery. *Nano Energy* ***2022***, 98, 107220.

[37] Duan, G.-S.; Wang, Y.; Luo, B.; Sun, L.-L.; Zheng, S.-N.; Huang, J.-Y.; Ye, Z.-Z. Taurine-mediated dynamic bridging strategy for highly stable Zn metal anode. *Energy Stor. Mater.* ***2023***, 61, 102882.

[38] Cao, H.; Huang, X.-M.; Li, Y.-X.; Liu, Y.; Zheng, Q.-J.; Huo, Y.; Zhao, R.-Y.; Zhao, J.-X.; Lin, D.-M. Regulating the solventized structure to achieve highly reversible zinc plating/stripping for dendrite-free Zn anode by sulfolane additive. *Chem. Eng. J.* ***2023***, 455, 140538.

[39] Wang, N.-Z.; Chen, X.; Wan, H.-Z.; Zhang, B.; Guan, K.-L.; Yao, J.; Ji, J.; Li, J.-Y.; Gan, Y.; Lv, L.; Tao, L.; Ma, G.-K.; Wang, H.-B.; Zhang, J.; Wang, H. Zincphobic Electrolyte Achieves Highly Reversible Zinc-Ion Batteries. *Adv. Funct. Mater.* ***2023***, 33, 2300795.

[40] Zhou, W.-J.; Chen, M.-F.; Quan, Y.-H.; Ding, J.; Cheng, H.-L.; Han, X.; Chen, J.-Z.; Liu, B.; Shi, S.-Q.; Xu, X.-W. Stabilizing zinc deposition through solvation sheath regulation and preferential adsorption by electrolyte additive of lithium difluoro (oxalato) borate. *Chem. Eng. J.* ***2023***, 457, 141328.

[41] Zhao, K.; Fan, G.-L.; Liu, J.-D.; Liu, F.-M.; Li, J.-H.; Zhou, X.-Z.; Ni, Y.-X.; Yu, M.; Zhang, Y. -M.; Su, H.; Liu, Q.-H. Cheng, F.-Y. Boosting the Kinetics and Stability of Zn Anodes in Aqueous Electrolytes with Supramolecular Cyclodextrin Additives. *J. Am. Chem. Soc.* ***2022***, 144, 10301−10308.

[42] Ouyang, K.-F.; Li, F.; Ma, D.-T.; Wang, Y.-Y.; Shen, S.-C.; Yang, M.; Qiu, J.-M.; Wen, W.-T.; Zhao, N.; Mi, H.-W.; Zhang, P.-X. Trace-Additive-Mediated Hydrophobic Structure Editing of Aqueous Zinc Metal Batteries for Enabling All-Climate Long-Term Operation. *ACS Energy Lett.* ***2023***, 8, 5229−5239.

[43] Zhu, J.-C.; Yang, M.; Hu, Y.; Yao, M.-J.; Chen, J.; Niu, Z.-Q. The Construction of Binary Phase Electrolyte Interface for Highly Stable Zinc Anodes. *Adv. Mater.* ***2023***, 2304426.


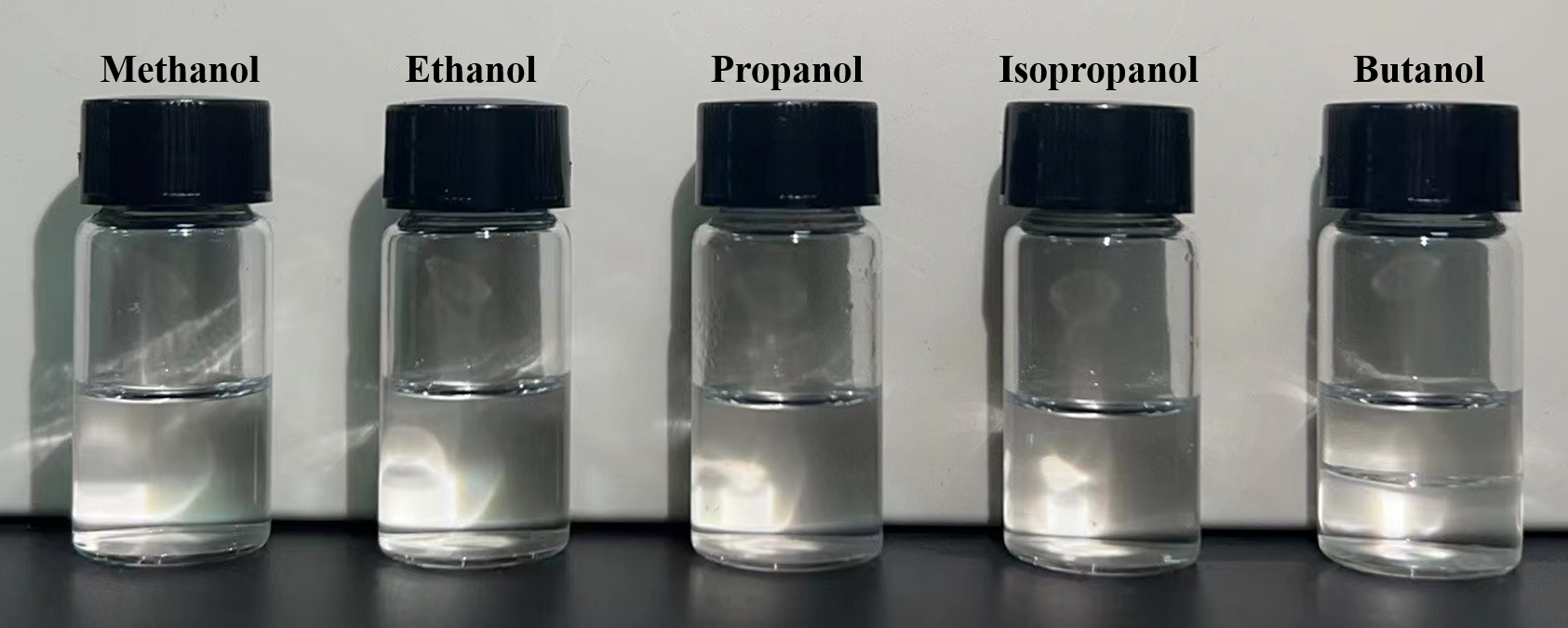


**Figure S1.** Optical photos of mixed solutions of various monohydric alcohols and water.


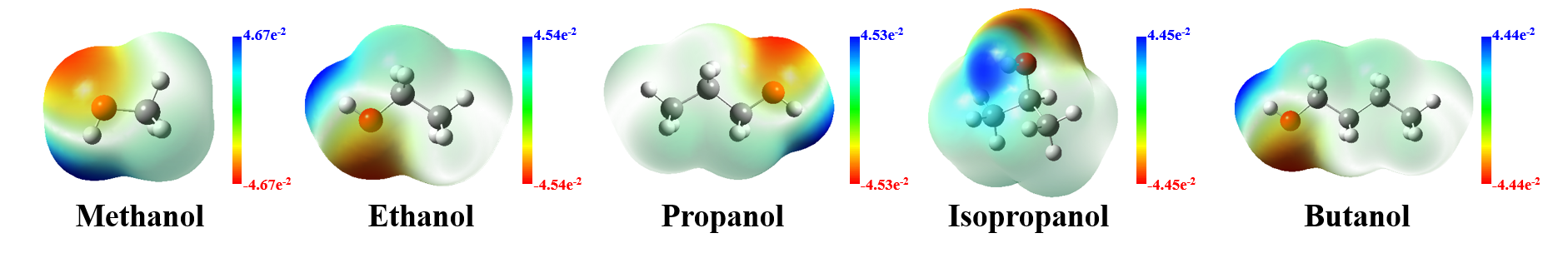


**Figure S2.** ESP distribution for various monohydric alcohols molecule obtained from DFT calculations.


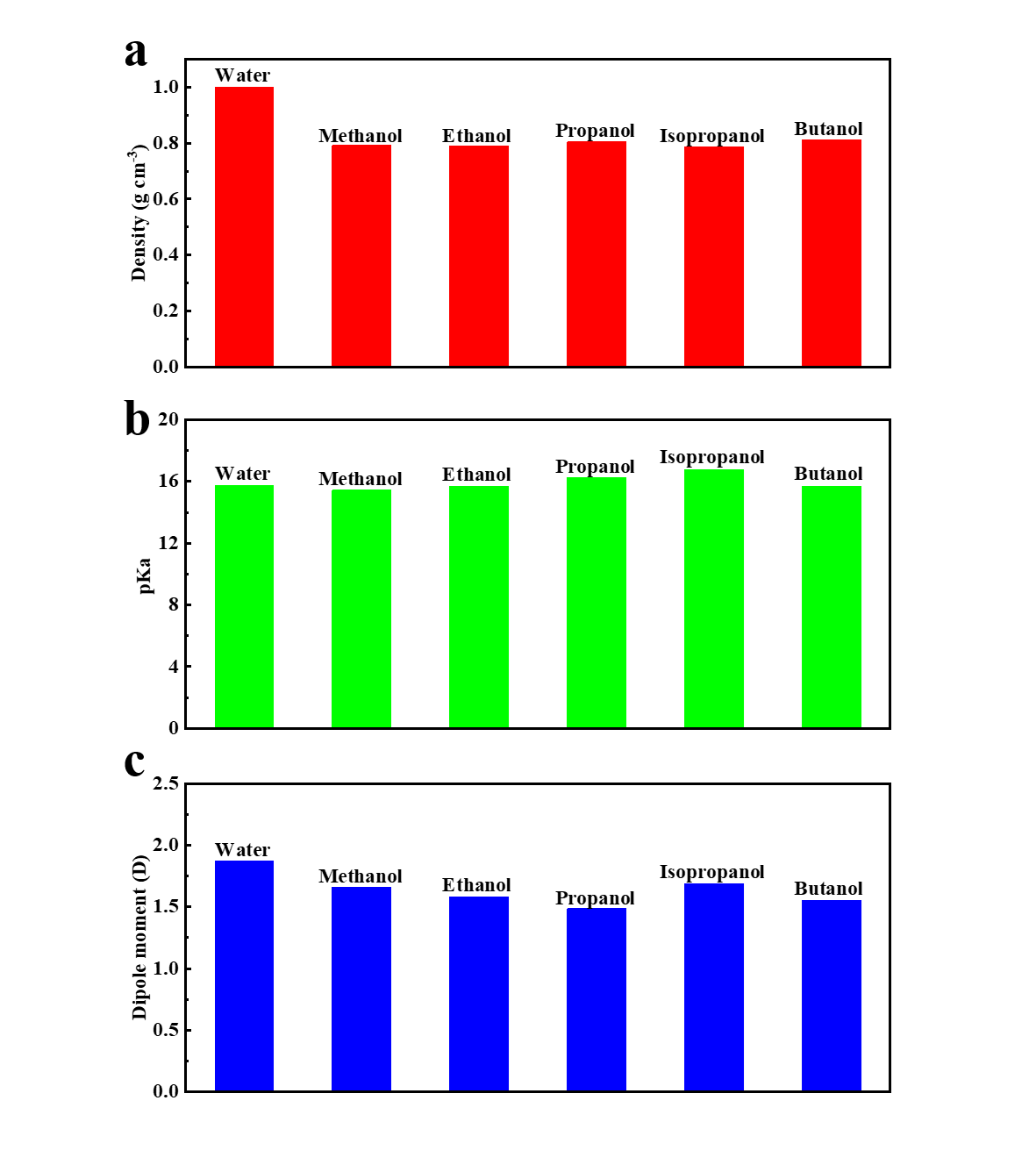


**Figure S3.** (a) Materials density, (b) pKa values and (c) dipole moment of various monohydric alcohols.


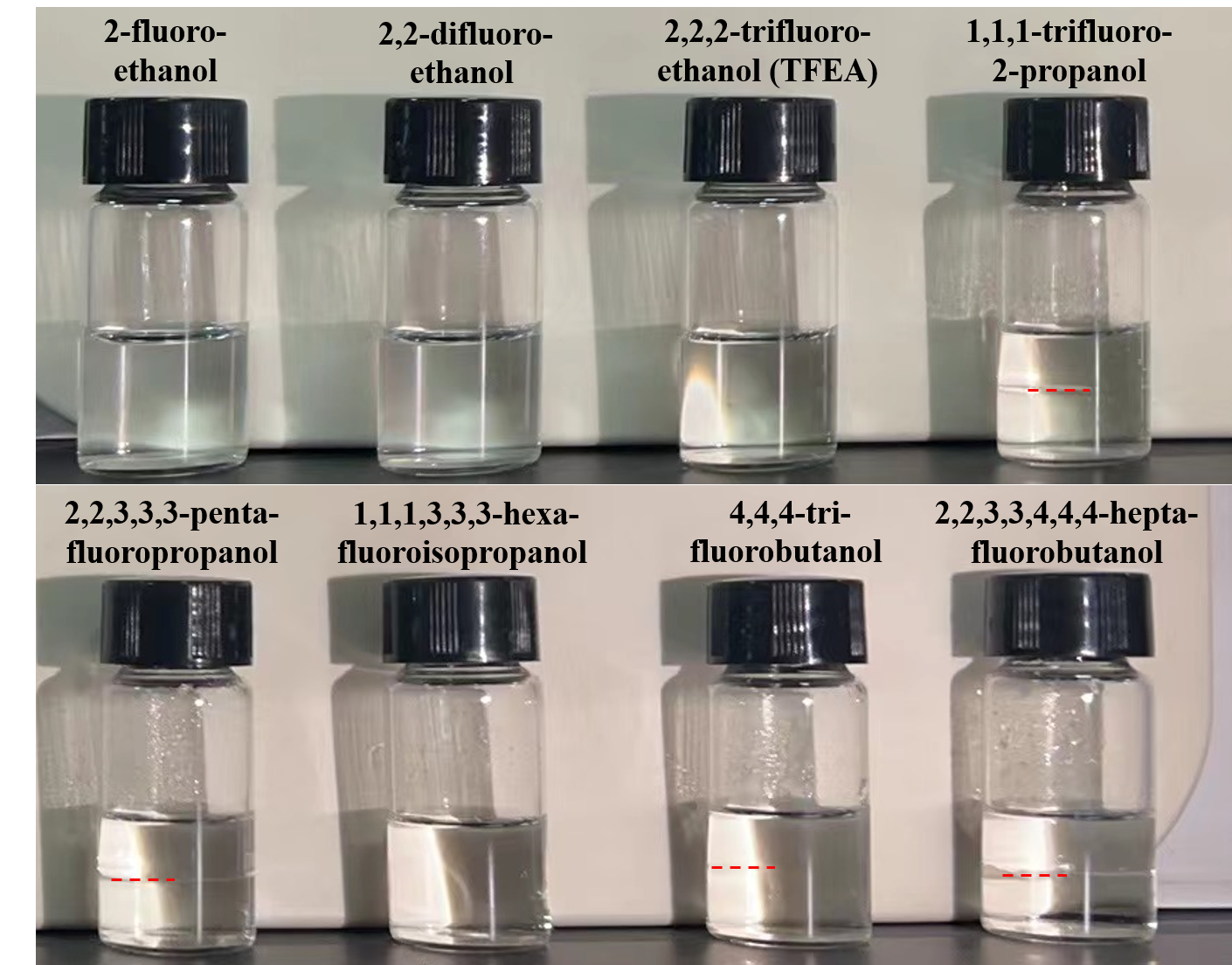


**Figure S4.** Optical photos of mixed solutions of various monohydric alcohols with C-F bond and water.


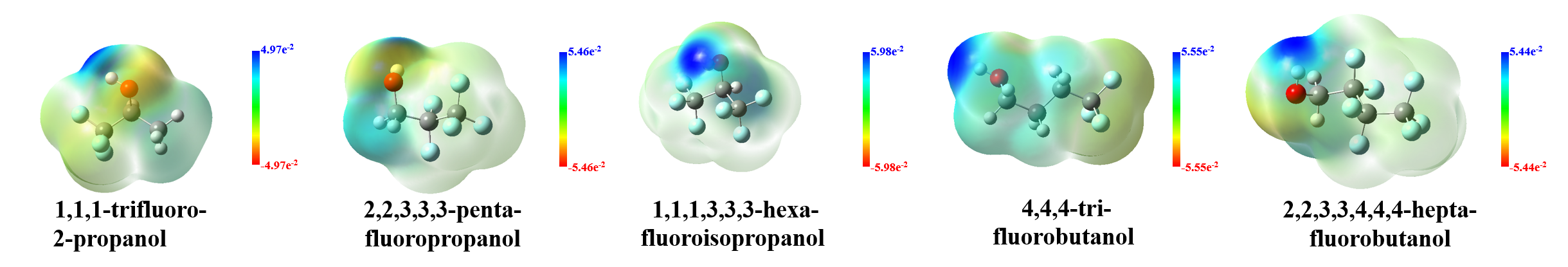


**Figure S5.** ESP distribution for various monohydric alcohols molecule with C-F bond obtained from DFT calculations.

**
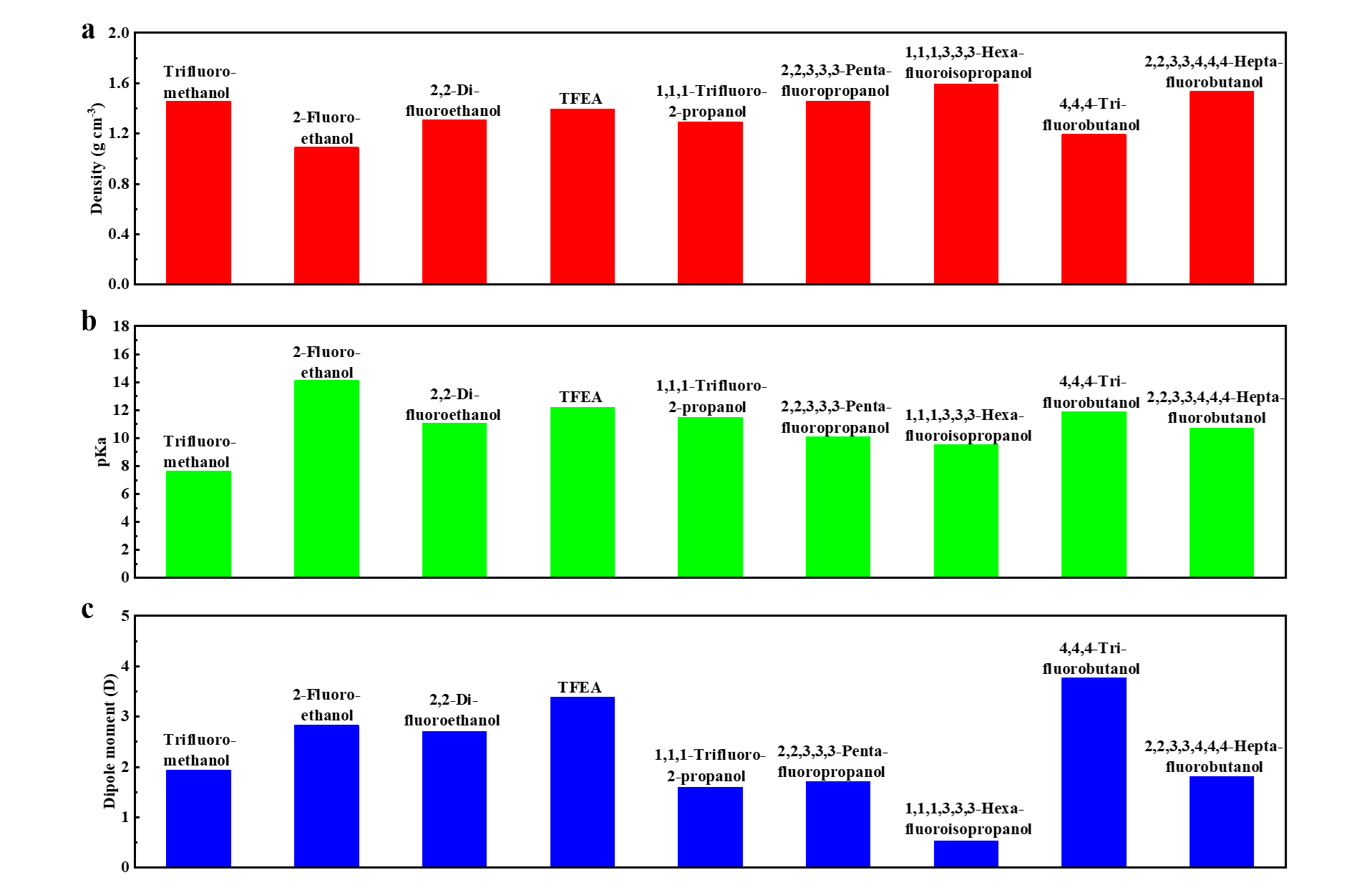
**

**Figure S6.** (a) Materials density, (b) pKa values and (c) dipole moment of various monohydric alcohols with C-F bond.


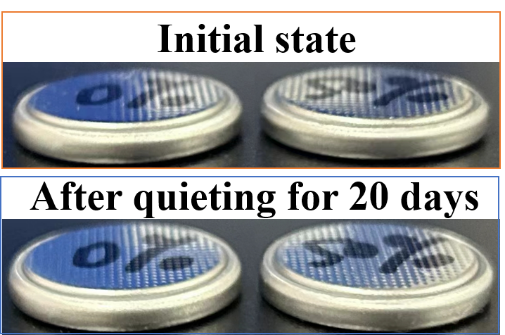


**Figure S7** Optical images of Zn//Zn symmetric cells with 0%-TFEA and 50%-TFEA at the initial stage and quieting for 20 days.


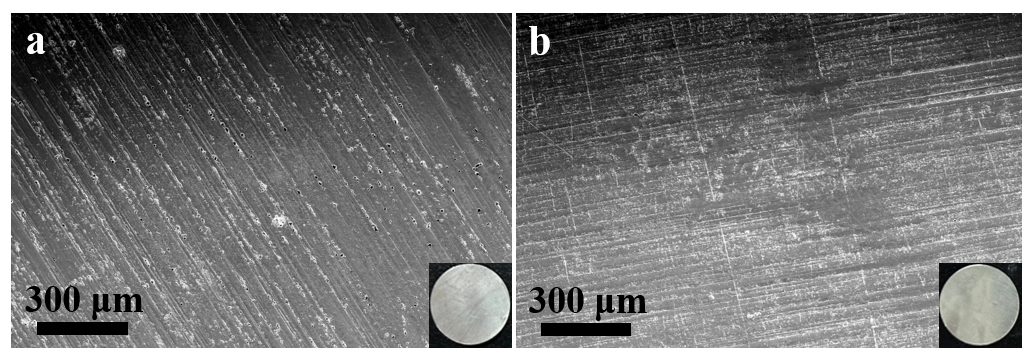


**Figure S8** SEM images and optical images (The insets) of Zn anodes soaking in (a) 0%-TFEA and (b) 50%-TFEA for 20 days.


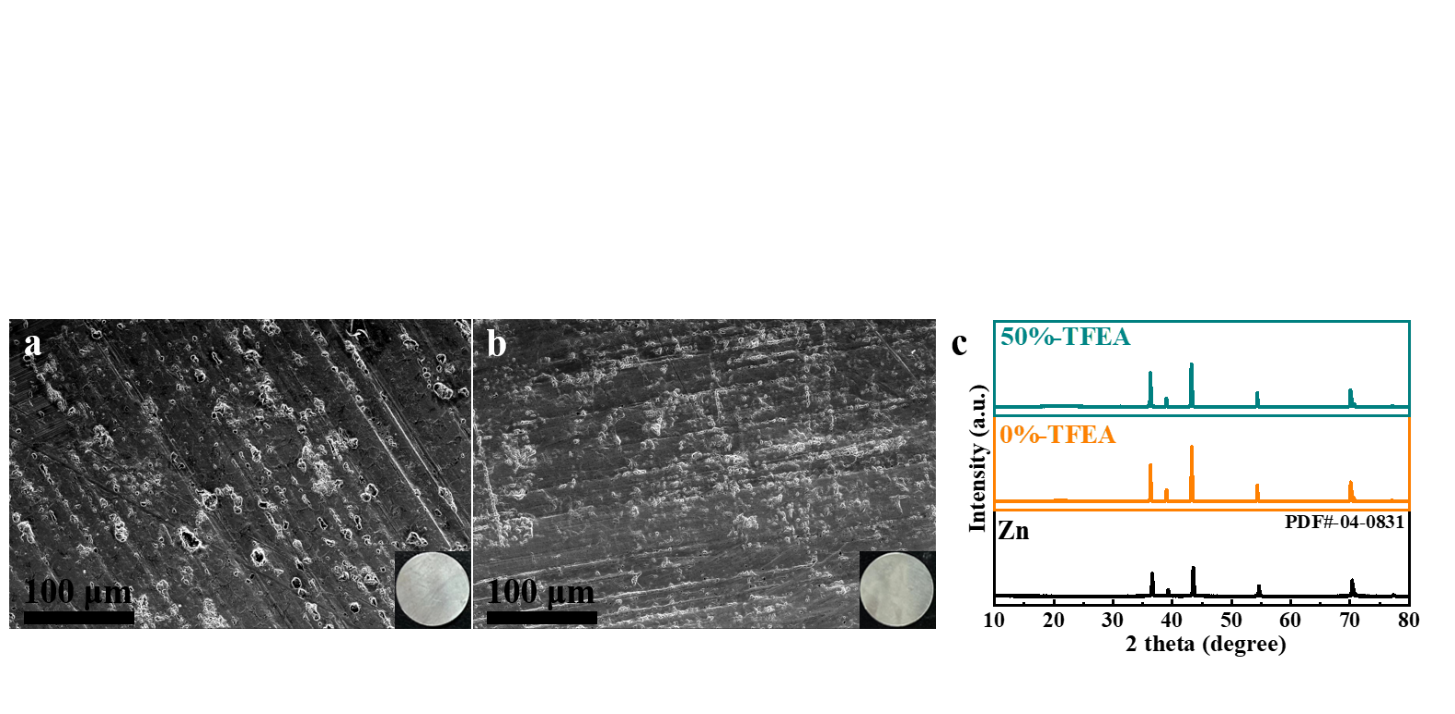


**Figure S9** XRD patterns of Zn anodes soaking in 0%-TFEA and 50%-TFEA for 20 days.


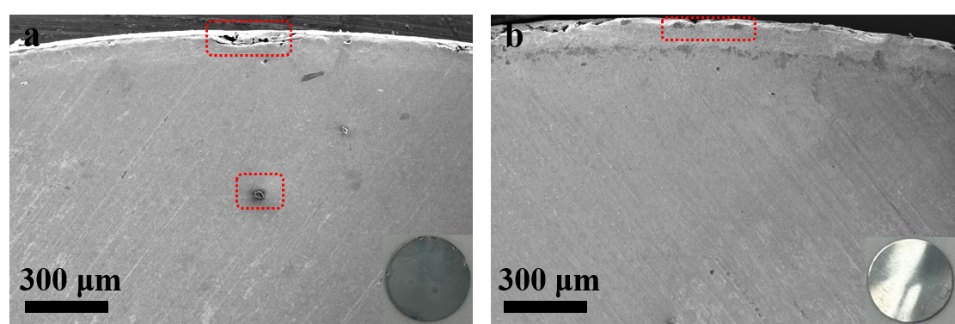


**Figure S10** SEM images and optical images (The insets) of Zn anodes soaking in (a) 0%-TFEA + 0.01 M CF_3_SO_3_H and (b) 50%-TFEA + 0.01 M CF_3_SO_3_H for 20 days.


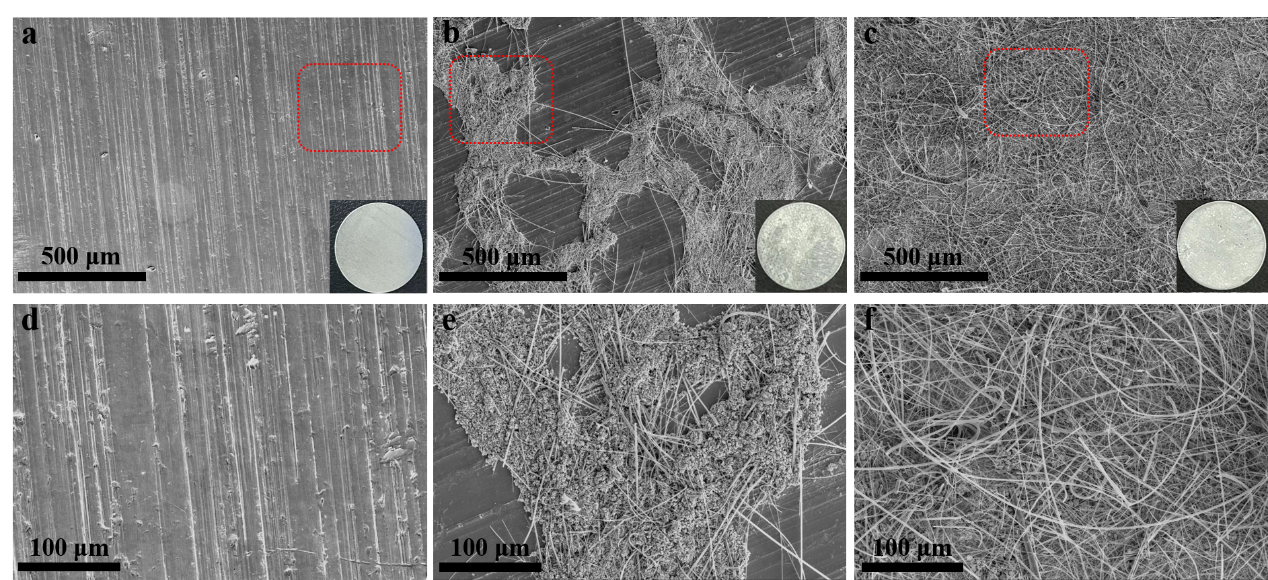


**Figure S11.** SEM images of Zn anode without deposition (a) and depositing in 0%-TFEA (b) and 50%-TFEA (c) at 10 mA h cm^−2^ (The insets are the optical images of Zn anode). (d-f) High resolution images of the red areas respectively.


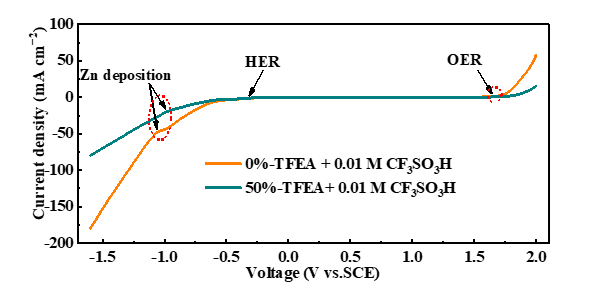


**Figure S12** Linear sweep voltammetry (LSV) curves of electrolytes by a three-electrode device.


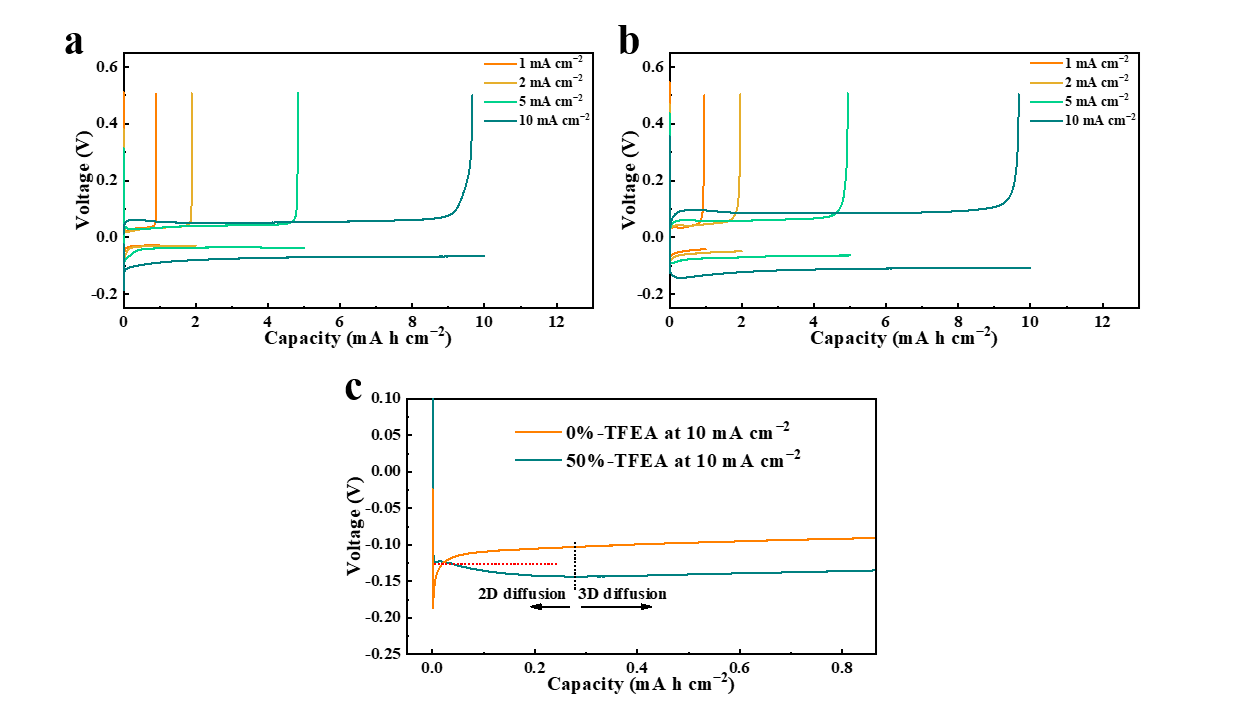


**Figure S13.** Charge-discharge curves of Cu//Zn half batteries with (a) 0%-TFEA and (b) 50%-TFEA at different current density. (c) Enlarged view of Charge-discharge curve of Cu//Zn half cells at 10 mA cm^−2^.


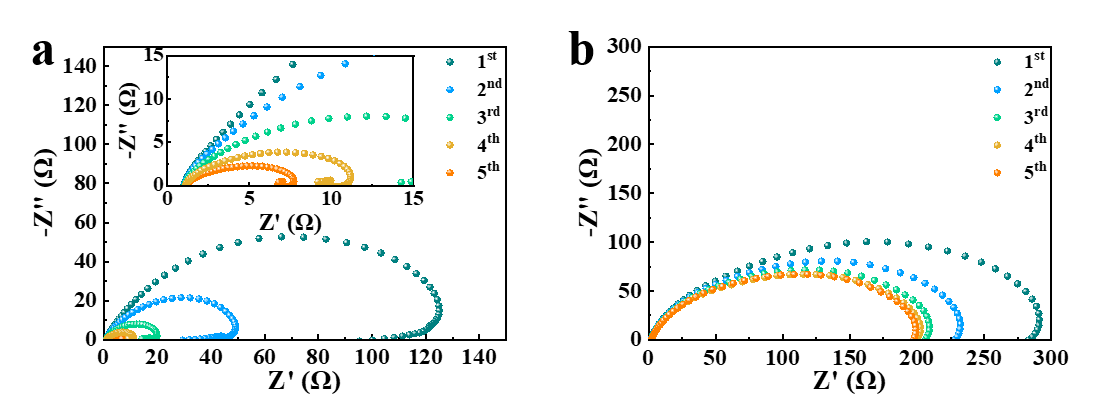


**Figure S14.** EIS plots of Cu//Zn half cells with (a) 0%-TFEA and (b) 50%-TFEA.


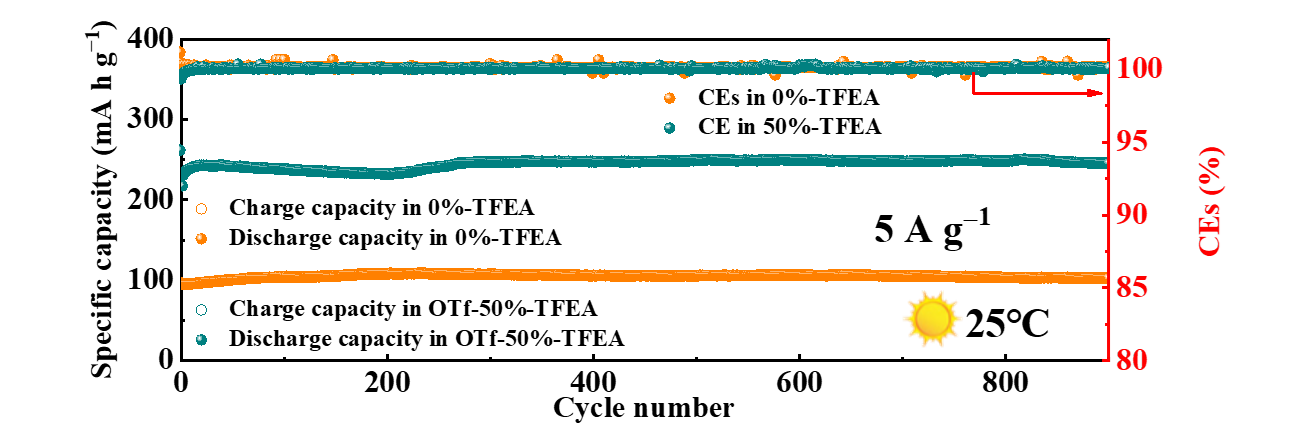


**Figure S15.** Cycle performance of V_2_O_5_ full batteries with (a) 0%-TFEA and (b) 50%-TFEA at 5 A g^−1^ at 25 ℃.


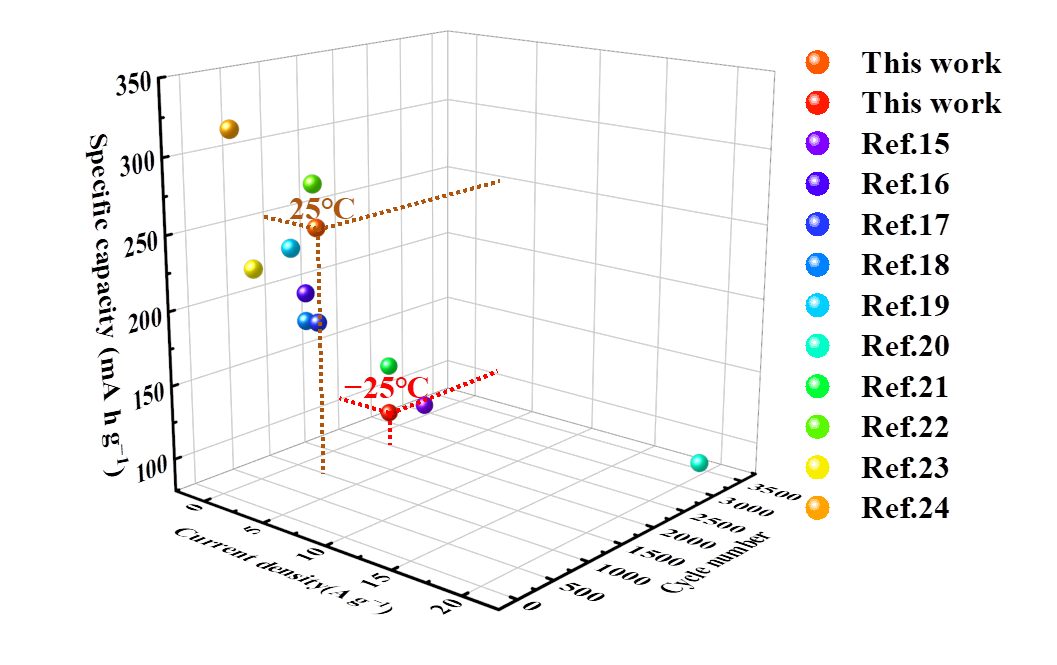


**Figure S16.** Comparison with reported electrolytes for ZIBs in terms of performance metrics.


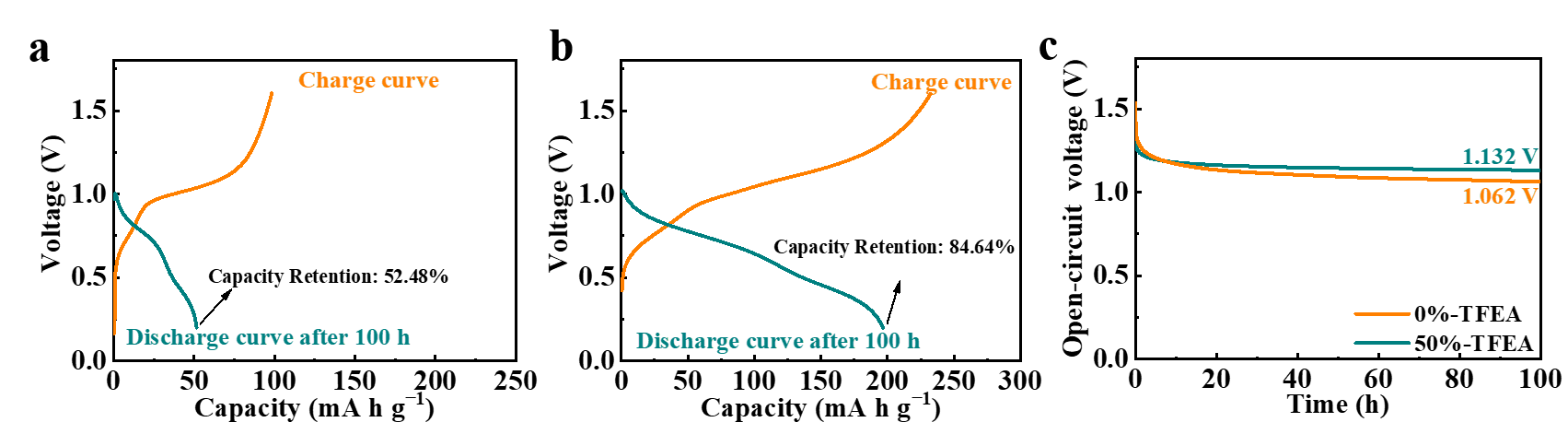


**Figure S17.** Charge-discharge curves of V_2_O_5_//Zn full batteries with (a) 0%-TFEA and (b) 50%-TFEA at 5 A g^−1^ at 25 ℃. (c) Open-circuit voltage curves of V_2_O_5_//Zn full batteries during 100 h.


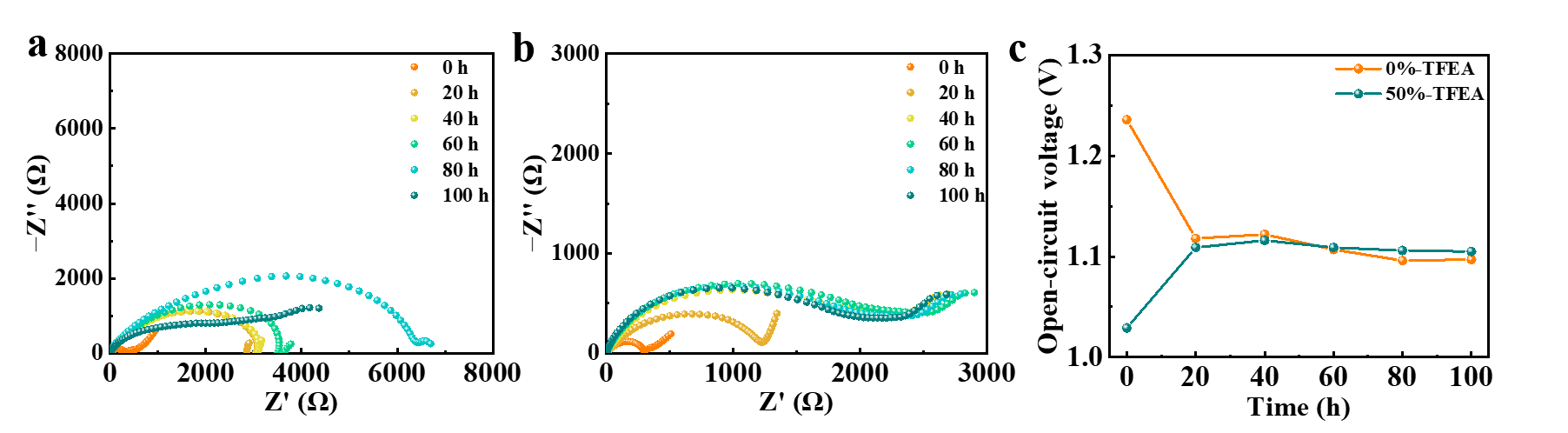


**Figure S18.** Nyquist plots of (a) 0%-TFEA and (b) 50%-TFEA and (c) the tested open-circuit voltage.


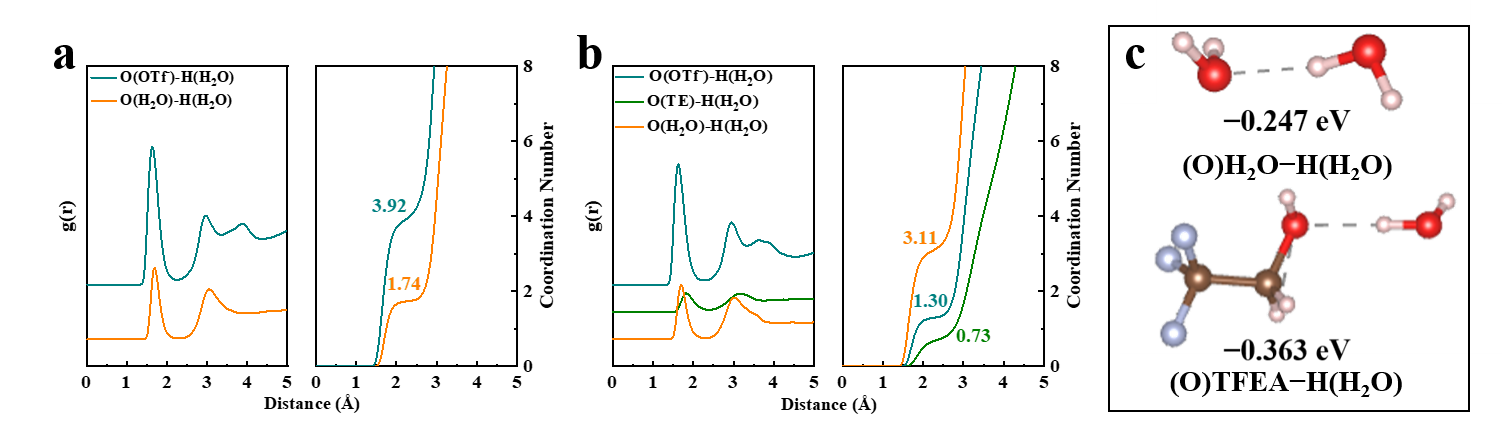


**Figure S19.** Radial distribution function and coordination number of H-bonds with H_2_O structures in (a) 0%-TFEA and (b) 50%-TFEA. (c) The binding energies of (O)H_2_O–(H)H_2_O and (O)TFEA–(H)H_2_O.


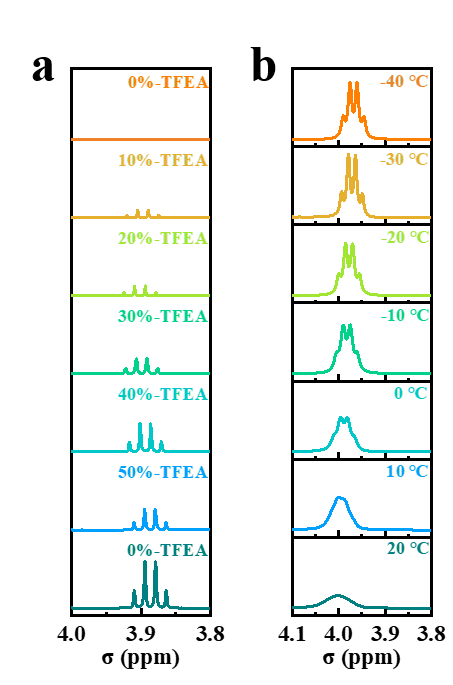


**Figure S20.**  (a) 1H NMR spectra of different electrolytes and (b) temperature-dependent 1H NMR resonance of 50%-TFEA.


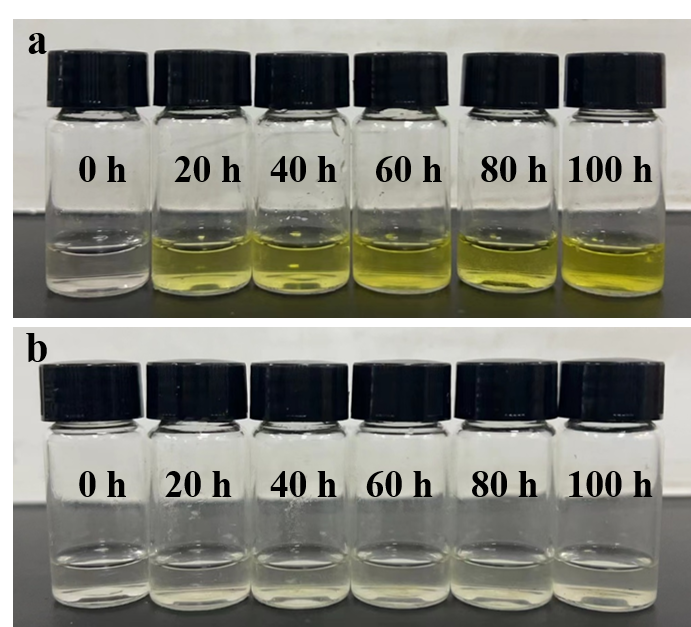


**Figure S21.** Optical photos of (a) 0%-TFEA and (b) 50%-TFEA, which have soaked V_2_O_5_ cathodes for different times.


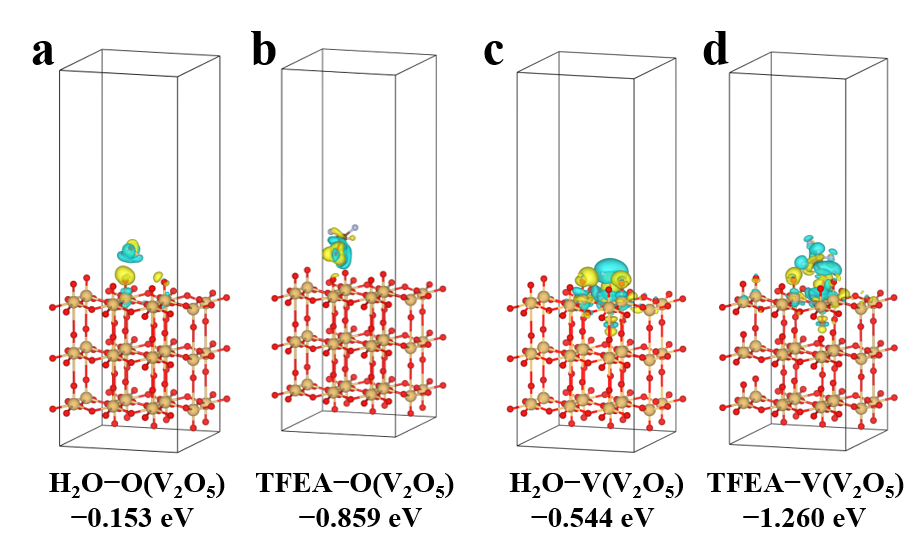


**Figure S22.** Adsorption energies of (a, c) H_2_O or (b, d) TFEA molecule around unsaturated O and V atoms in V_2_O_5_.


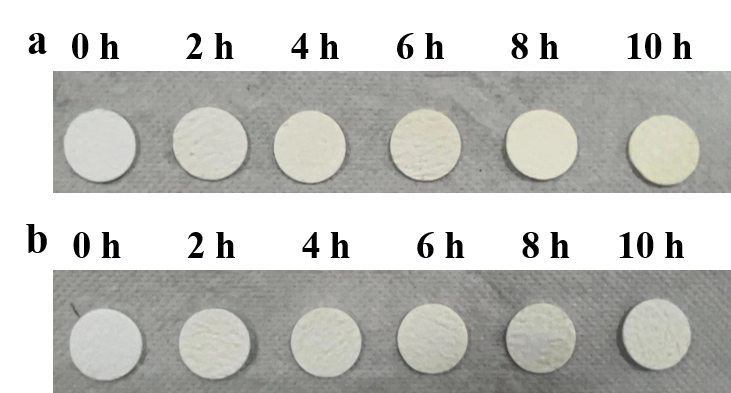


**Figure S23.** Optical photos of separators in V_2_O_5_//Zn full batteries with (a) 0%-TFEA and (b) 50%-TFEA after different working times.


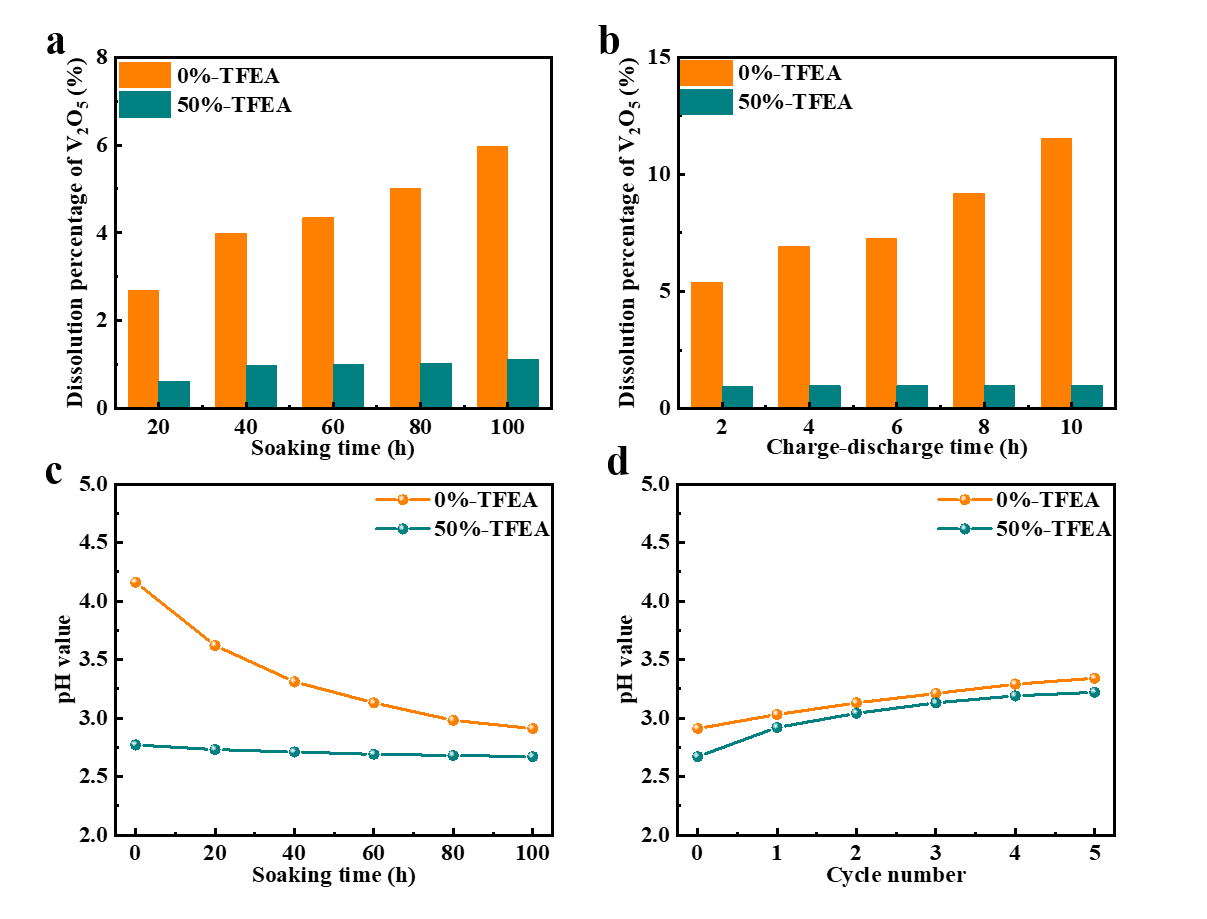


**Figure S24.** Concentrations of V element after (a) soaking and (b) charge-discharge times in different electrolytes by ICP. pH values of 0%-TFEA and 50%-TFEA after (c) soaking V_2_O_5_ cathode for different times and (d) after different charge-discharge cycles.

**
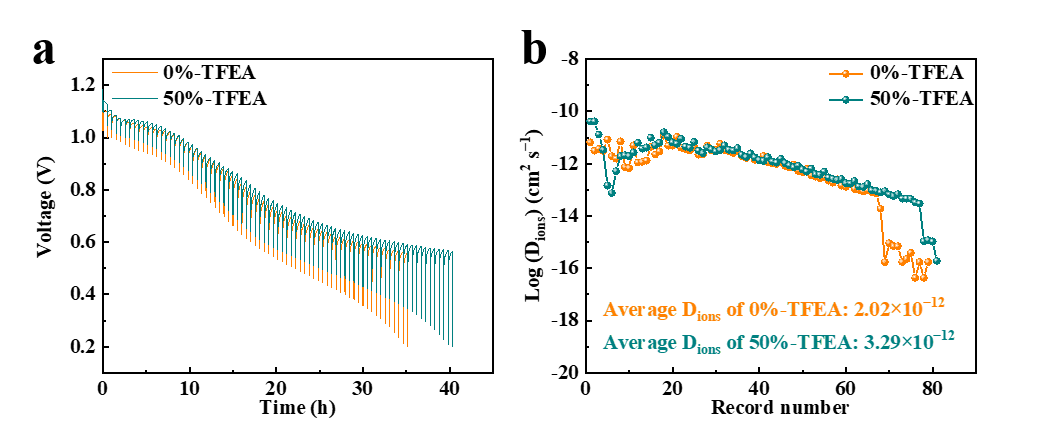
**

**Figure S25.** GITT curves and calculated ion diffusion coefficients of (a) 0%-TFEA and (b) 50%-TFEA.

**
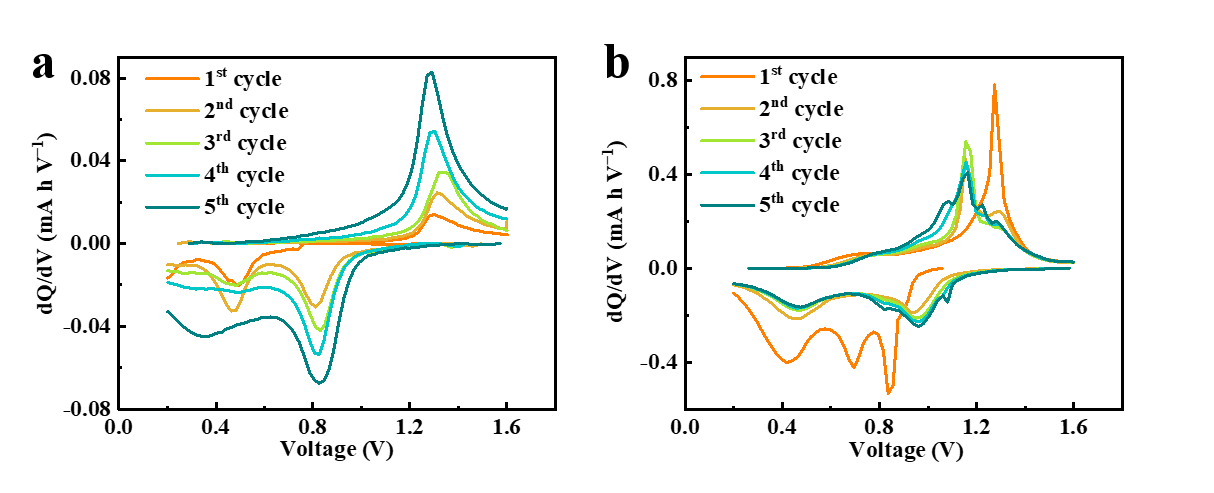
**

**Figure S26.** Differential capacity curves of V_2_O_5_//Zn full batteries with (a) 0%-TFEA and (b) 50%-TFEA.


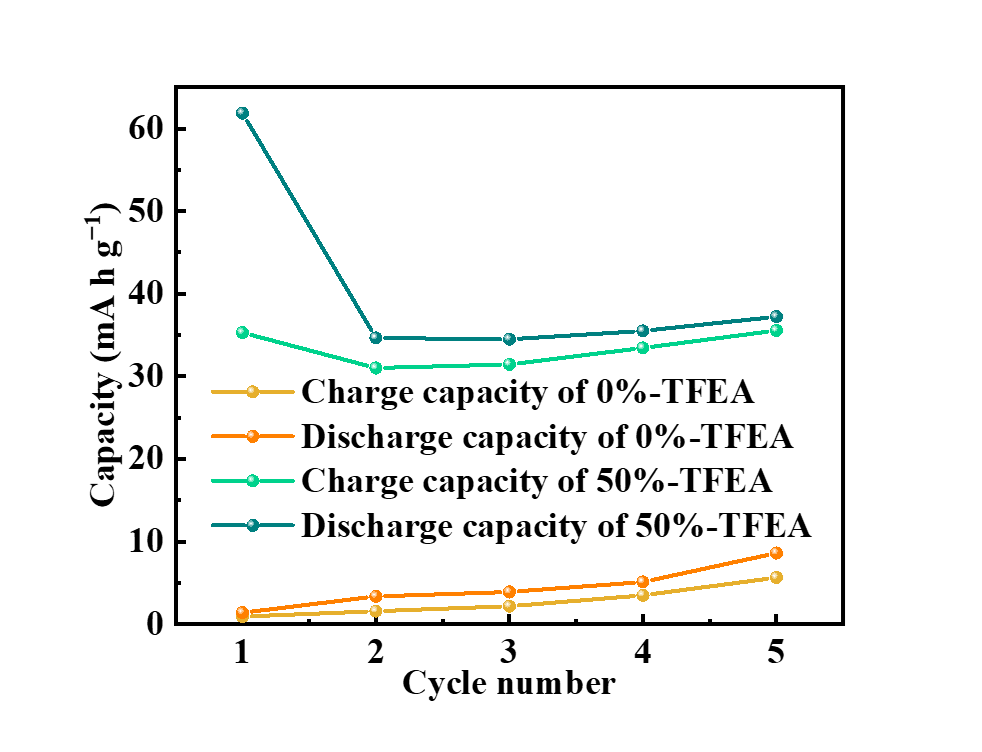


**Figure S27.** Charge and discharge capacity of V_2_O_5_//Zn full with 0%-TFEA and 50%-TFEA at first five cycles of activation.

**
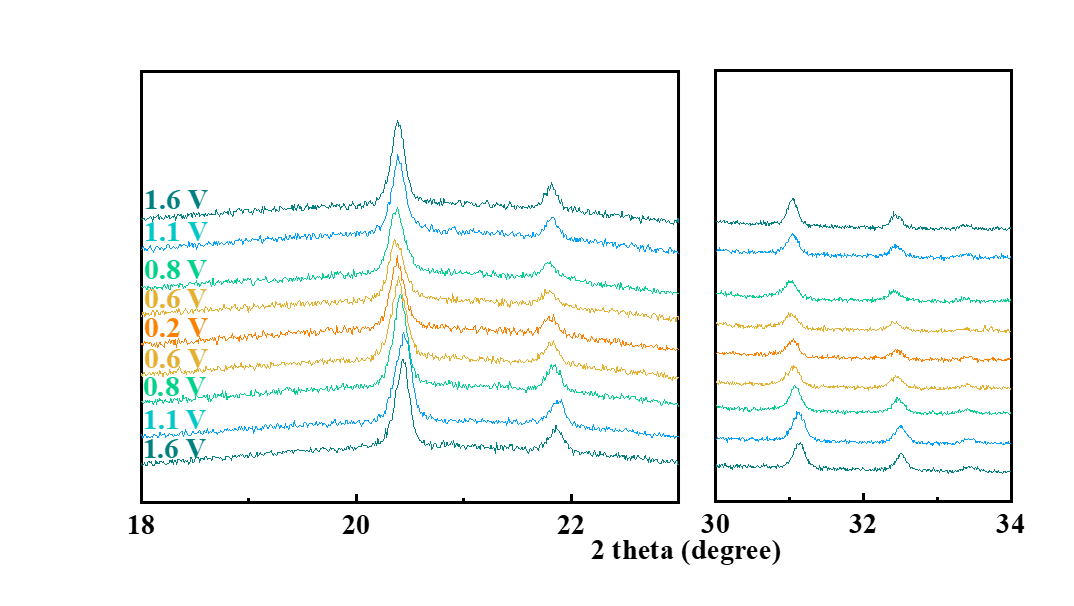
**

**Figure S28.** *Ex-site* XRD patterns of V_2_O_5_ cathode in 0%-TFEA.

**
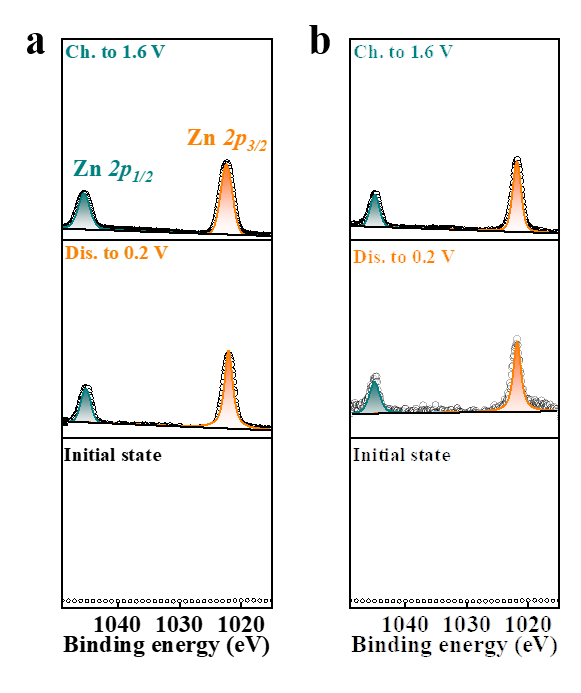
**

**Figure S29.** XPS spectra of Zn *2p* orbitals of V_2_O_5_ cathodes in (a) 0%-TFEA and 50%-TFEA.

**
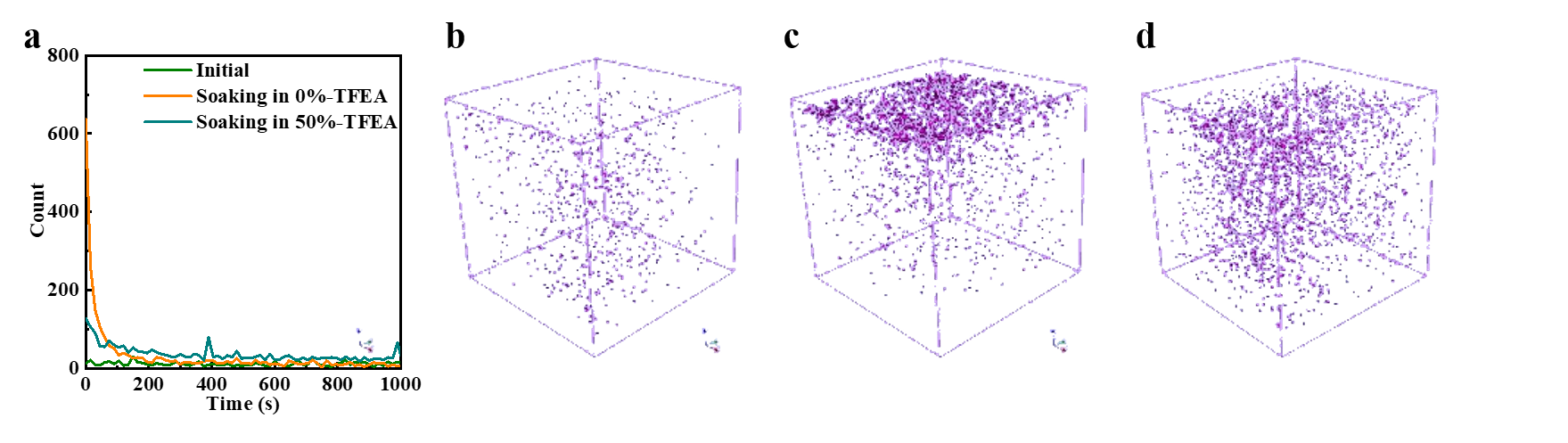
**

**Figure S30.** (a) Positive mode of [CF_3_]^+^ fragments and its corresponding 3D variation of ToF-SIMS intensity of (b) initial state, (c) 0%-TFEA, and (d) 0%-TFEA.

**Video S1.** Wettability test of V_2_O_5_ cathodes.
